# KGG: Knowledge-Guided Graph Self-Supervised Learning to Enhance Molecular Property Predictions

**DOI:** 10.1021/acs.jcim.5c01068

**Published:** 2025-09-08

**Authors:** Van-Thinh To, Phuoc-Chung Van Nguyen, Gia-Bao Truong, Tuyet-Minh Phan, Tieu-Long Phan, Rolf Fagerberg, Peter F. Stadler, Tuyen Ngoc Truong

**Affiliations:** † Faculty of Pharmacy, 249295 University of Medicine and Pharmacy at Ho Chi Minh City, 41 Dinh Tien Hoang, District 1, Ho Chi Minh City 700000, Vietnam; ‡ Bioinformatics Group, Department of Computer Science, Interdisciplinary Center for Bioinformatics, and School for Embedded and Composite Artificial Intelligence (SECAI), Leipzig University, Härtelstraße 16−18, D-04107 Leipzig, Germany; § Department of Mathematics and Computer Science, 6174 University of Southern Denmark, DK-5230 Odense M, Denmark; ∥ Max Planck Institute for Mathematics in the Sciences, Inselstraße 22, D-04103 Leipzig, Germany; ⊥ Department of Theoretical Chemistry, 9180 University of Vienna, Währingerstraße 17, A-1090 Vienna, Austria; # Facultad de Ciencias, Universidad Nacional de Colombia, Bogotá D.C. 111321, Colombia; ∇ Center for Non-coding RNA in Technology and Health, University of Copenhagen, Ridebanevej 9, DK-1870 Frederiksberg, Denmark; ○ Santa Fe Institute, 1399 Hyde Park Rd., Santa Fe, New Mexico 87501, United States

## Abstract

Molecular property prediction has become essential in accelerating advancements in drug discovery and materials science. Graph Neural Networks have recently demonstrated remarkable success in molecular representation learning; however, their broader adoption is impeded by two significant challenges: (1) data scarcity and constrained model generalization due to the expensive and time-consuming task of acquiring labeled data and (2) inadequate initial node and edge features that fail to incorporate comprehensive chemical domain knowledge, notably orbital information. To address these limitations, we introduce a Knowledge-Guided Graph (KGG) framework employing self-supervised learning to pretrain models using orbital-level features in order to mitigate reliance on extensive labeled data sets. In addition, we propose novel representations for atomic hybridization and bond types that explicitly consider orbital engagement. Our pretraining strategy is cost efficient, utilizing approximately 250,000 molecules from the ZINC15 data set, in contrast to contemporary approaches that typically require between two and ten million molecules, consequently reducing the risk of potential data contamination. Extensive evaluations on diverse downstream molecular property data sets demonstrate that our method significantly outperforms state-of-the-art baselines. Complementary analyses, including t-SNE visualizations and comparisons with traditional molecular fingerprints, further validate the effectiveness and robustness of our proposed KGG approach. The key advantages of KGG are its data efficiency and architectural versatility, driven by orbital-informed representations. By distilling essential chemical knowledge from modest corpora, it avoids extensive pretraining and excels in low-data fine-tuning, providing a robust and chemically meaningful foundation for diverse GNN architectures.

## Graphical abstract

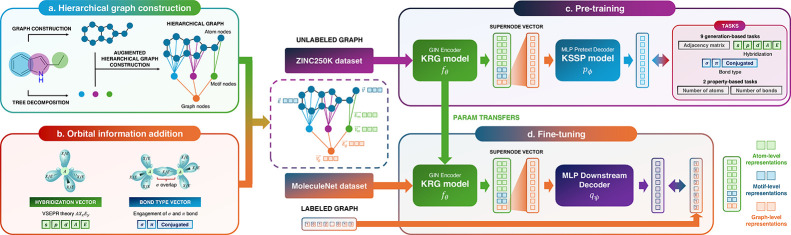

## 1 Introduction

Significant advancements in Artificial Intelligence (AI) have profoundly influenced the drug discovery sector, primarily through the implementation of machine learning and deep learning techniques. These computational methods, renowned for their ability to process and analyze large volumes of data with remarkable speed and precision, have demonstrated significant potential to enhance efficiency and reduce costs across various stages of the drug development pipeline, such as uncovering drug–target interactions,^1,2^ designing and optimizing drug structures,^3–5^ and predicting 3D structures of proteins.^6^ The identification of molecules with desired properties, in particular bioactivity and toxicity, remains one of the major interests in the field of Computer-Aided Drug Design (CADD) and Drug Development.^7–9^ To this end, molecular property prediction maps a molecule (typically encoded as a SMILES string or a molecular graph) to a target chemical property, yielding either a continuous value for regression tasks (e.g., solubility or logP) or a discrete label for classification tasks (binary or multiclass, e.g., toxicity or bioactivity), thereby enabling efficient virtual screening, lead optimization, and toxicity assessment.

Molecular representations play a pivotal role in accurately predicting molecular properties, serving as the foundation for computational models to capture the essential structural and chemical features of molecules.^10–12^ The choice of molecular representation profoundly affects the performance of predictive algorithms, as a well-chosen representation helps ensure that the model can generalize across diverse chemical spaces while maintaining interpretability. Molecular descriptors,^13–15^ including chemical and physical properties of compounds, and molecular fingerprints^16–18^ that encode the structure and properties of molecules into binary vectors are frequently used as input features in predictive models.

Graph-based deep learning has garnered significant attention within the artificial intelligence community,^19–22^ driven primarily by the ubiquity of graph-structured data across various domains, including e-commerce,^23^ transportation,^24^ and chemistry.^25^ Chemical structures inherently adopt graph representations, making graph-based models particularly promising for molecular representation learning.^26,27^ Despite their notable successes in supervised and semisupervised learning scenarios, these models heavily depend on manually labeled data, leading to several limitations: (1) the acquisition and annotation of large-scale labeled data sets can be prohibitively expensive, particularly in specialized fields such as chemistry and medicine,^28^ as well as in fields where data sets are very extensive, such as in the study of social and citation networks;^29^ (2) supervised models frequently suffer from limited generalization and increased susceptibility to overfitting, particularly when labeled data is scarce;^30^ and (3) the accuracy and reliability of labels significantly affect model performance, making these methods vulnerable to label noise and uncertainty.^31^ These inherent challenges highlight the necessity for developing alternative methodologies capable of reducing the dependency on labeled data while maintaining robust and generalizable performance.

Despite the persistent scarcity of labeled data, the abundance of large-scaled unlabled data set, particularly in chemistry,^32^ represents an invaluable resource, contingent upon effective utilization. Self-Supervised Learning (SSL) emerges as a promising paradigm in situations where extensive unlabeled data exist but only limited labeled data, exist. In practice, SSL models are pretrained using sizable unlabeled data sets through various pretext tasks, thereby capturing general representations of the underlying data manifold. Subsequently, these pretrained models undergo fine-tuning using much smaller labeled data sets to optimize task-specific performance. Recent investigations into SSL methodologies for molecular representation learning,^33–41^ which are summarized in Supporting Section 2.2, have demonstrated impressive performance and robustness across diverse benchmark data sets, detailed in Tables S1 and S2. Notably, the *Hierarchical Molecular Graph Self-Supervised Learning* (HiMol) framework introduced by Zang et al.,^33^ which leverages hierarchical graph structures to facilitate integrated representation learning, and the *Knowledge-guided Pretraining of Graph Transformer* (KPGT) model by Li et al.,^41^ which employs line graph representations complemented by a central node aggregating and propagating chemical properties, have been identified as state-of-the-art approaches exhibiting remarkable robustness and predictive power.

Although such significant advancements have been achieved in molecular representation learning, several critical challenges persist: (1) molecular graphs predominantly encode atom-level information within nodes and edges but generally omit orbital details, which are a crucial factor underlying chemical valence bonds, as well as other chemical properties, thereby limiting representation expressivity; (2) one-hot encoding strategies are computationally demanding for large-scale data sets^42^ and insufficient in capturing meaningful relations among categorical variables,^43^ such as bond types or orbital hybridization; (3) the excessive reliance on the pretraining data sets utilized by SSL models may lead to data contamination.^44^ Such contamination can inadvertently incorporate test instances into the training process, thereby artificially enhancing the measured generalization performance.

To address the aforementioned challenges, we introduce a novel graph-based SSL framework, Knowledge-Guided Graph (KGG), which explicitly integrates orbital information into molecular graphs. Our approach is inspired by the work of Benkö et al.,^45^ which employed *orbital graphs* where hybridization-aware atomic orbitals are represented as vertices and where the corresponding edges, interpreted as orbital overlaps, depict localized chemical bonds responsible for chemical reactions. The proposed KGG model consists of two essential components: (1) the Knowledge Representation Graph (KRG) architecture, serving as an encoder to extract hierarchical graph representations enriched with orbital-level chemical insights; and (2) the Knowledge Self-Supervised Pretraining (KSSP) multitask pretext module, designed for comprehensive pretraining. This module encompasses tasks ranging from adjacency matrix reconstruction to the prediction of molecular attributes, notably orbital hybridization and bond characteristics. Taken together, this enables KGG to address the drawbacks mentioned above of conventional molecular graph representations. We furthermore restrict our pretraining data to mitigate the risk of data contamination, as suggested by Jiang et al.,^44^ thereby ensuring more robust capabilities across diverse data sets.

## 2 Results and Discussion

### 2.1 KGG Framework

The KGG model is an SSL framework designed to encode molecular representations by utilizing knowledge vectors as initial features (Figure 1). Its architecture comprises two primary components: KSSP and the KRG. The KSSP operates as a pretraining decoder driven by pretext tasks aimed at reconstructing orbital knowledge vectors, adjacency matrices, and two fundamental molecular properties, using a Multi-Layer Perceptron (MLP) which receives graph embeddings extracted by KRG. By jointly optimizing these tasks, the model effectively captures rich orbital and molecular information, allowing the KRG encoder to learn meaningful representations that enhance the overall predictive performance (Figure 1c). The KRG, built upon the Graph Isomorphism Networks (GIN) architecture,^19^ functions as a graph embedding extractor by processing hierarchical graphs and incorporating *orbital* knowledge vectors as inputs to produce graph embeddings (Figure 1c).

**Figure 1:**
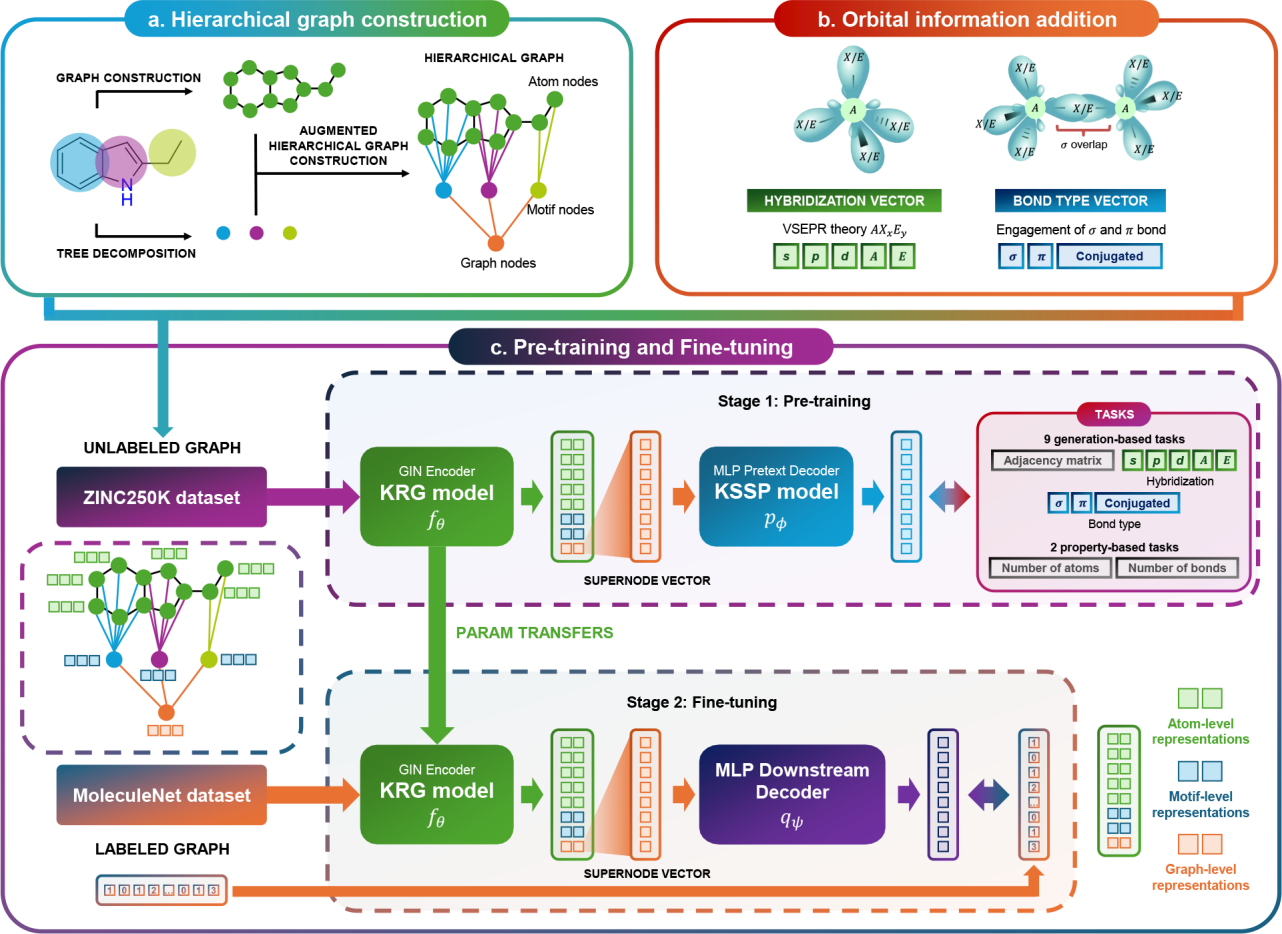
Overview of the KGG framework. (a) The hierarchical molecular graph construction involves three critical steps: (1) establishing an atom-level foundational graph, (2) decomposing this graph into motif-level nodes, and (3) integrating a supernode that encapsulates the entire molecular structure, thereby creating a comprehensive hierarchy with atom-, motif-, and graph-level representations. (b) Initial feature embedding highlights the unique integration of orbital-level chemical information. Atom features are derived based on *hybridization* and Valence Shell Electron Pair Repulsion (VSEPR) theory, while bond features distinctly represent relationships characterized by the count of σ and π bonds, and existence of conjugation system (δ). (c) Training procedure of KGG comprises two stages: pretraining with 11 pretext tasks in the KSSP module, followed by fine-tuning of the pretrained KRG model for downstream evaluations on MoleculeNet benchmarks.

### 2.2 Molecular Property Predictions

Our KGG model demonstrated superior performance over 13 state-of-the-art (SOTA) SSL approaches, details of which are provided in Supporting Sections 2.1 and 2.2 for classification and regression tasks on data sets from MoleculeNet, respectively.

Regarding the classification tasks, our KGG model attained the highest average ROC-AUC score (75.5 ± 0.3) across six data sets, as depicted in Figure 2a. Detailed results obtained from three independent experiments using distinct random seeds are presented in Table S1. We further validated the superior performance of KGG through statistical testing, *t test* in particular, which confirmed a statistically significant improvement (*p*-value <0.01) relative to the second-best method (see Figure 2d). For a more comprehensive view of individual data set performance and corresponding statistical assessments, we refer to Figures S1 and S2, respectively. Specifically, KGG delivered superior results on BACE and SIDER, with ROC-AUC scores of 86.3 ± 0.2 and 64.9 ± 1.0, respectively. On BACE, KGG significantly outperformed the second-best model (HiMol, 84.3 ± 0.3) at a *p*-value <0.001. Likewise, on SIDER, KGG (64.9 ± 1.0) significantly exceeded the second-best approach (MGSSL, 61.8 ± 0.8) with *p*-value <0.05. Although KGG ranked second on BBBP (72.5 ± 0.7) after HiMol (73.2 ± 0.8), the difference did not reach statistical significance (*p*-value >0.05). Overall, these findings highlight the predictive capability of KGG compared to that of extant SSL methods, underscoring the practical utility of orbital information in molecular representation learning. Compared to the previously state-of-the-art HiMol,^33^ KGG achieved an average ROC-AUC improvement of 2.4% and outperformed in four out of six data sets (BACE, SIDER, ToxCast, ClinTox).

**Figure 2:**
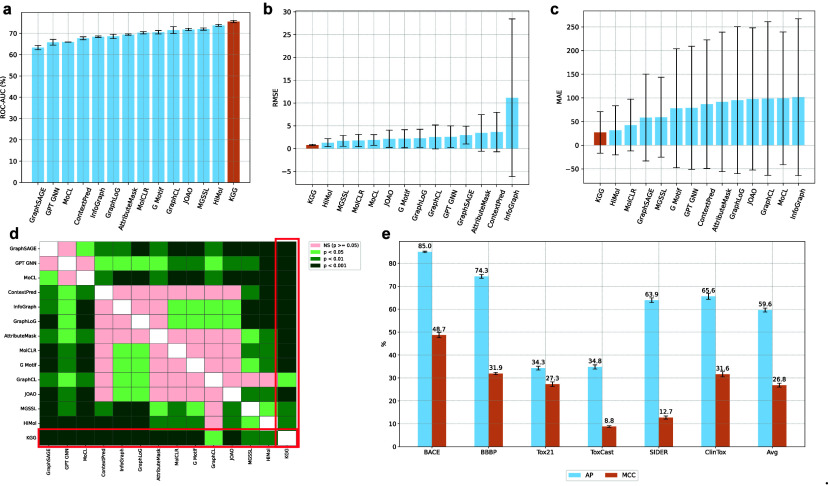
(a) The average results of KGG and other SSL approaches on six classification data sets from MoleculeNet are measured in terms of ROC-AUC (%). The results, presented as mean ± standard deviation, were obtained through three independent runs, each utilizing a different random seed. (b) The average RMSE values across three regression physicochemical data sets ESOL, FreeSolv, and Lipophilicity of KGG and previous SSL methods. (c) The average MAE values on three regression quantum data sets QM7, QM8, QM9 of KGG and other SSL methods. (d) The *t* test analysis compares the performance of KGG against other SSL methods upon six classification data sets. The *t* test results indicate that the performance of our KGG model is statistically significant superior to other SSL methods, with p value at three levels: 0.05, 0.01, and 0.001. (e) The Average Precision (AP) and Matthews Correlation Coefficient (MCC) values on six classification data sets from MoleculeNet of KGG.

Among the spectrum of high-performing SSL methods, motif-based architectures, including KGG, HiMol,^33^ G_Motif,^34^ and MGSSL,^36^ have consistently exhibited superior predictive ability. Indeed, five of the six top-performing models in our experiments employ explicit motif representations, thereby emphasizing the pivotal role of motif information in molecular representation learning. Motifs, conceived as functional fragments encapsulating key chemical features, enable these models to detect and exploit domain-specific patterns inherent in molecular structures. Within this elite subset of motif-based approaches, KGG surpassed its counterparts in four of the six benchmark data sets, underscoring the utility of explicitly incorporating orbital information into motif-based designs. Departing from previous SSL paradigms,^33–36,40,41,46–52^ KGG encodes molecular representations via a KRG “backbone,” which merges motif structures with orbital knowledge vectors in the initial feature space for both nodes and edges. These enhanced descriptors, incorporating hybridization states and bond types (see Section 4.2), strengthen the model’s expressive power, ultimately leading to more accurate molecular structure learning and property predictions.

Turning to regression tasks, Figure 2b and c illustrates the average root-mean-square error (RMSE) and mean absolute error (MAE) achieved across six data sets in MoleculeNet. As recommended by MoleculeNet,^53^ MAE serves as the evaluation metric for quantum-mechanical data sets (QM7, QM8, QM9), while RMSE is used for physicochemical data sets (ESOL, FreeSolv, Lipophilicity). Table S2 and Figure S3 provide a detailed comparison between KGG and other SSL architectures on each respective regression data set. Notably, KGG delivered the best overall performance, achieving an average RMSE of 0.78 ± 0.15 and an average MAE of 27.076 ± 43.86. While we do not report *T*-test results here due to the limited number of data sets (three in each category), KGG nevertheless outperformed its peers on ESOL, QM8, and QM9, achieving scores of 0.731, 0.665, and 77.684, respectively (see Figure S3).

Compared to HiMol, which is the prior SOTA model, KGG reduced the average RMSE by 39% and outperformed on two of three individual RMSE benchmarks. The average MAE was likewise reduced by 14%, with KGG again surpassing HiMol on two of the three MAE data sets. Notably, KGG attained a 68% lower RMSE than HiMol on FreeSolv and yielded a 15% reduction in MAE on QM7, a quantum-mechanical data set often considered among the most challenging (see Table S2). These findings underscore the effectiveness of integrating orbital knowledge with motif-centric representations, thereby enabling KGG to capture intricate chemical relationships and deliver a robust predictive performance.

Furthermore, we evaluated the computational efficiency of the KGG model, confirming its viability for large-scale applications (see Table S12). While the one-time pretraining on vast chemical libraries like ZINC15 and ChEMBL29 takes approximately 12 and 109 h, respectively, the model can be rapidly fine-tuned for specific tasks, such as for QM9 in under 2 h. Crucially, with an inference speed of only 1 ms per compound, KGG is well-suited for high-throughput virtual screening of multibillion molecule libraries.

### 2.3 Comparisons with Traditional Fingerprints

We performed a comparative analysis to evaluate the representational capacity of the KGG fingerprint, derived from the *graph-level representations* produced by the KGG model, against three widely used conventional fingerprints: MACCS, ECFP4, and RDK7. Our evaluation revealed two key findings: first, a kNN classifier, selected for its simplicity, trained on KGG fingerprints, consistently achieves higher predictive accuracy compared to classifiers utilizing conventional fingerprints; second, t-distributed stochastic neighbor embedding (t-SNE) visualization clearly demonstrates that KGG fingerprints yield significantly improved clustering of data points, highlighting their enhanced discriminative ability.

Specifically, KGG fingerprints outperform traditional fingerprints on the BACE and BBBP data sets for *classification* tasks (Figure 3a) as well as on the ESOL, FreeSolv, Lipophilicity, and QM7 data sets for *regression* tasks (Figure 3b). Importantly, these improvements are consistently observed under both *random* and *scaffold* splitting strategies, indicating that the observed superiorities are fundamentally attributable to our knowledge vectors, particularly the incorporation of orbital information. Moreover, the notable predictive performance achieved even with a simple algorithm such as kNN suggests that KGG fingerprints inherently capture richer and more chemically meaningful information than traditional fingerprints, independent of the complexity of the predictive model. This inference is further substantiated by comparable predictive results when employing either kNN or MLP classifiers on KGG fingerprints, as presented in Figure S4.

**Figure 3:**
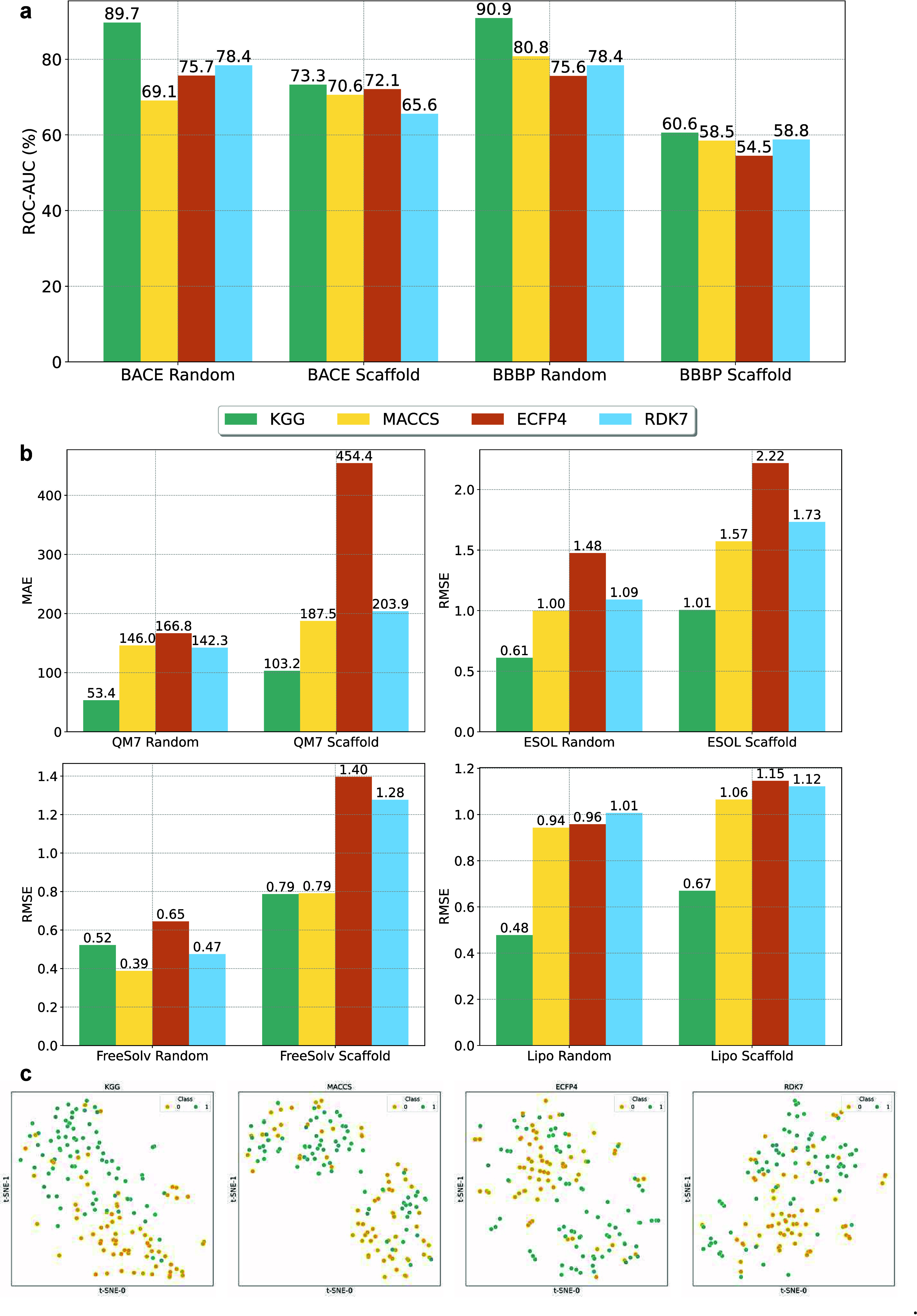
A comparison of classification and regression performance of KGG, MACCS, ECFP4, and RDK7 fingerprints using random and scaffold splitting strategies, as well as t-SNE visualizations. (a) The ROC-AUC values for two classification data sets (BACE and BBBP). (b) RMSE and MAE values for four regression data sets (ESOL, FreeSolv, Lipophilicity, QM7). (c) t-SNE visualization of the validation BACE test set for each fingerprint type.

Furthermore, we applied t-SNE visualization to directly compare KGG fingerprints with conventional alternatives, as illustrated in Figure 3c. The visualization distinctly demonstrates superior class separation in the BACE data set when using KGG fingerprints, as opposed to conventional fingerprints. A similar pattern is consistently observed across other data sets, as further detailed in Figures S5, S6, S7, S8, S9, and S10. This clear separation reaffirms the strong capability of our KGG model in generating effective graph neural network fingerprints for molecular representation, enabling various downstream tasks.

Building on these findings, we further examined the chemical coherence of our motif embeddings by visualizing the learned chemical space via t-SNE projections (see Supporting Section 3.5). The resulting 2D projections of the global motif vectors are presented in Figures S12, S13, and S14. For data sets centered on biological activity and physical properties, we observe distinct, label-consistent clusters. This clustering demonstrates that KGG effectively distills chemically meaningful information into its motif-level representations, enabling robust discrimination between different functional classes across diverse molecular data sets. In the quantum-mechanical data sets (QM7, QM8, and QM9), however, the embeddings form a more continuous distribution, reflecting the intrinsically complex and smoothly varying nature of quantum properties rather than discrete class boundaries.

### 2.4 Ablation Study

To assess the effectiveness of each component in our proposed KGG model, we performed a series of ablation experiments. By selectively removing key modules from the KGG architecture, we created several model variants, as detailed in Supporting Section 3.4, that highlight the contribution of each component.^54^ The outcomes of these experiments are summarized in Figure 4, Table S4, and Table S5.

**Figure 4:**
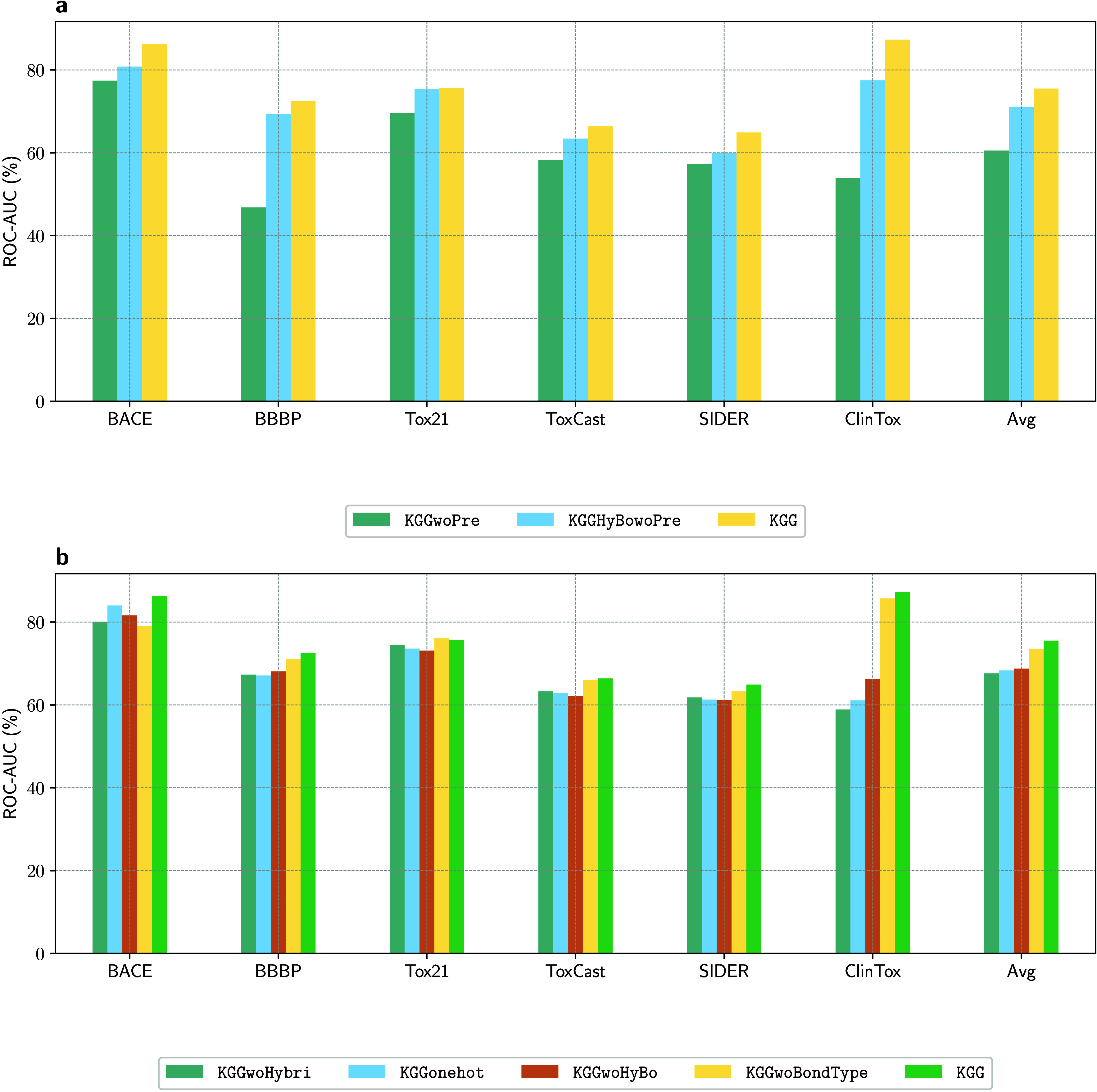
Ablation study of the KGG variants. (a) ROC-AUC values for six classification data sets across different KGG pretraining configurations, with the final three columns indicating their overall average. (b) Performance of six classification data sets under KGG variants that use different featurization approaches, where the last five columns show the average ROC-AUC values for these variants.

We performed a detailed investigation of the impact of the pretraining on the performance of our KGG model, as depicted in Figure 4a. Specifically, we compared three configurations: without pretraining (KGGwoPre), with partial pretraining excluding orbital-oriented tasks (KGGHyBowoPre, retaining only reconstruction tasks for adjacency matrices, number of atoms, and number of bonds), and the fully pretrained KGG. Our results indicate that KGGwoPre consistently yields the lowest performance across six classification data sets, underscoring the necessity of effective pretraining for robust molecular representations. Meanwhile, KGGHyBowoPre improves significantly over KGGwoPre, yet the complete KGG model demonstrates a superior performance, emphasizing the critical contribution of orbital-oriented tasks. Thus, integrating these tasks in self-supervised learning enhances weight initialization and facilitates more effective model adaptation.

To evaluate the influence of graph representations on downstream fine-tuning performance, we systematically examined four variants of the KGG model (Figure 4b): KGGonehot (utilizing only one-hot encodings), KGGwoHyBo (omitting both hybridization and bond type vectors), KGGwoBondType (omitting bond type vectors), and KGGwoHybri (omitting hybridization vectors). Our analyses underscore the pivotal role of atomic hybridization, evidenced by KGGwoBondType’s consistent outperformance over KGGwoHybri, thereby highlighting that *hybridization* provides more predictive utility compared to bond type alone, which may introduce unnecessary noise. Notably, the complete KGG model, incorporating both *hybridization* and *bond type* vectors, demonstrated superior performance relative to those of both KGGwoBondType and KGGwoHybri. This finding aligns chemically with the fundamental principle that molecular bonding involves hybrid orbital overlaps, underscoring the synergy between *hybridization* and *bond type* descriptors. Additionally, reliance exclusively on simplistic categorical encodings (KGGonehot) resulted in suboptimal performance compared with other models, highlighting their inadequacy in capturing nuanced chemical information. Consistent with these ablation findings, a random-masking analysis (Supporting Section 3.7) confirms that model performance is more sensitive to the loss of hybridization states than bond types, underscoring the chemical primacy of local atomic geometry. The sharpest performance decline occurs when masking both, which confirms their synergistic value. Remarkably, even with the simultaneous masking of all node and edge features, the model’s predictive power degrades gracefully (retaining 67.4% ROC-AUC and 1.0 RMSE). This resilience demonstrates that KGG’s hierarchical message-passing architecture effectively distributes and preserves chemical information within higher-order motif and graph-level representations, compensating for the loss of explicit local descriptors. Ultimately, our fully integrated KGG model, combining comprehensive orbital-related features, consistently delivered the strongest performance, validating the significant advantages of embedding detailed chemical knowledge into molecular graph representations.

To probe the interplay between our chemically aware module and established graph learning frameworks, we integrated the KGG module into four distinct GNN backbones: GIN, GCN, GAT, and GraphSAGE (see Tables S6–S7). The results reveal a clear synergistic relationship: the KGG+GIN pairing proved most potent, achieving the highest classification ROC-AUC (75.5%) and lowest physicochemical regression RMSE (0.780) (see Figure 5), as GIN’s high expressive power effectively capitalizes on the rich features from our module. Conversely, the KGG+GAT combination underperformed, which we attribute to a conceptual redundancy between GAT’s explicit attention mechanism and the implicit chemical hierarchy already established by KGG. Notably, for predicting quantum properties, KGG+GraphSAGE achieved the lowest MAE (25.3), suggesting that its efficient aggregation of local atomic environments is particularly adept for tasks dominated by local chemical effects. Thus, our study demonstrates that KGG provides significant, architecture-dependent benefits, with maximal gains realized when the GNN’s inductive bias complements, rather than competes with, the chemically informed representations from our module.

**Figure 5:**
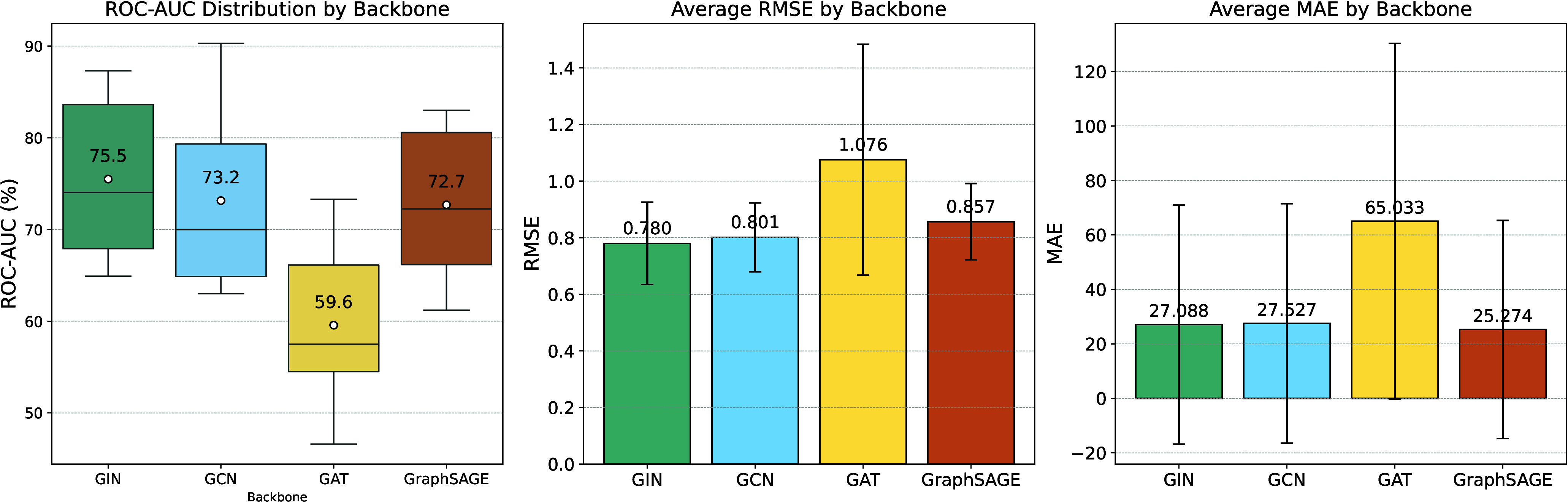
Performance of KGG with GIN, GCN, GAT, and GraphSAGE backbones on MoleculeNet benchmarks.

We next examined whether the model’s richer feature set confers robustness under two distinct scenarios: data scarcity during fine-tuning and corpus diversity during pretraining. First, to address data scarcity in fine-tuning, a critical challenge in many chemical applications, we progressively subsampled the training split (80% of the data in our fixed 8:1:1 train/valid/test split—to 70%, 60%, ..., down to 10% in 10-point steps), keeping hyperparameters fixed to test true generalization. The results (Table S8, Figure S11) demonstrate a graceful degradation of performance. Even with a 90% reduction in training examples, the mean classification ROC-AUC only fell from 75.5% to 59.4%, while regression errors saw a controlled increase (+0.52 RMSE, +19.7 MAE). This resilience underscores that by encoding fundamental chemical principles, KGG learns highly data-efficient representations, making it robust even when labeled data is limited.

Second, we investigated if pretraining on a larger, more functionally diverse chemical space would yield further benefits. To this end, we pretrained our model on the ChEMBL29 corpus (approximately two million molecules) and evaluated it on the same downstream tasks. Remarkably, the performance remained virtually unchanged compared to the model pretrained on ZINC15: the mean ROC-AUC across classification tasks decreased by a negligible 0.7 percentage points to 74.8%, and regression errors rose by a mere +0.028 RMSE and +0.633 MAE (Tables S9–S10). This stability strongly suggests that our orbital-reconstruction objective learns fundamental, transferable principles of electronic structure rather than simply memorizing statistical patterns of a specific chemical library. The lack of further improvement, despite the vast increase in data, likely reflects *objective saturation*:^55^ the finite set of hybridization and bond-type classes are already sufficiently represented within the smaller ZINC15 corpus, meaning additional data provides diminishing returns for this specific pretraining task.

Together, our comprehensive ablation studies demonstrate that (i) chemically informed hybridization and bond-type features are indispensable for peak accuracy, enabling the model to learn universal chemical principles that generalize across diverse chemical spaces rather than memorizing data set-specific motifs, and (ii) the resulting model maintains competitive performance under the low-data regimes typical of early stage drug discovery campaigns.

### 2.5 Data Contamination

Data contamination, defined as the fraction of molecules in downstream *test* splits that also occur in the pretraining corpus, has artificially boosted the performance of SSL models on downstream tasks.^44^ This phenomenon arises because the model merely memorizes the structures of test sets within the pretraining data set, rather than exhibiting robust generalization. Our KGG model outperformed other SSL methods while maintaining a low rate of data contamination and utilizing a significantly smaller pretraining data set comprising 250,000 samples, in contrast to the 2 to 10 million data points employed by others.

To rule out performance inflation from data comtamination in pretraining, we computed the *contamination* ratio for all benchmarks (see Table S11), including the potent KPGT model.^41^ KGG achieves the lowest contamination ratio while maintaining state-of-the-art downstream performance, demonstrating that its predictive power stems from learning fundamental physicochemical principles rather than scaffold memorization. A direct comparison with KPGT (Table 1, Figure 6) reveals two primary observations: (1) KGG exhibits an exceptionally low contamination rate (0.2%), contrasting sharply with the substantially higher rate observed in KPGT (81.7%); (2) despite this significantly lower contamination, KGG achieves competitive performance on diverse tasks, including classification (e.g., BACE, SIDER, ClinTox; see Figure 6b) and regression (e.g., ESOL, FreeSolv, Lipo; see Figure 6a). Notably, even when KPGT contamination is relatively moderate, such as 37.8% in ClinTox or 19.7% in BACE, the KGG model consistently matches or surpasses KPGT’s performance. This observation suggests that KPGT’s superior results on certain data sets may be influenced by memorization of test-set structures during pretraining, undermining fair comparative evaluation. Consequently, our results demonstrate that KGG maintains robust generalization capability and competitive downstream performance, despite a markedly lower level of data set contamination.

**Table 1:** Data Contamination Comparison between KGG and KPGT Models^a^

	Contaminated data points		Test data points		Cont. ratio (%) (↓**)**	
Task type	KGG	KPGT	KGG	KPGT	KGG	KPGT
Classification	6	1799	2289	2289	**0.3**	78.6
Regression	0	559	598	598	**0**	93.5
Overall	6	2358	2887	2887	**0.2**	81.7

a (↓) denotes that a smaller number is better.

**Figure 6:**
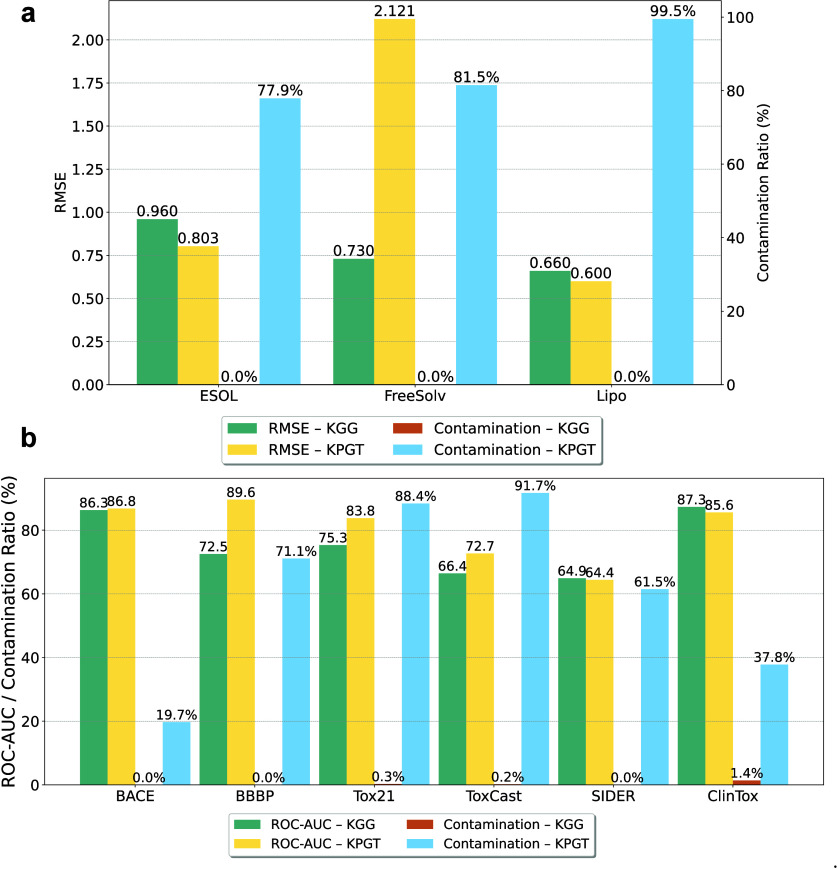
An analysis of data contamination for KGG and KPGT is presented. (a) The RMSE performance of the two models is assessed on three regression data sets, also including the corresponding contamination ratios. (b) The ROC-AUC performance of both models is evaluated on six classification data sets, with their respective contamination ratios reported alongside.

### 2.6 Limitations of Benchmarking Metrics

Binary classification constitutes a fundamental task of molecular property prediction in general and SSL in particular. Here, the area under the ROC curve (ROC-AUC) has conventionally been employed as the primary evaluation metric, recommended by MoleculeNet.^33–36,40,41,46–52^ Despite its widespread adoption, ROC-AUC captures only sensitivity and a false positive rate, neglecting positive and negative predictive values. This can lead to misleadingly optimiztic performance estimates, particularly in the context of class-imbalanced data sets. For example, in typical bioactivity screens with only ∼1% actives (*N*_*p*_ = 100, *N*_*n*_ = 9900), a model that ranks all 100 actives before retrieving 900 inactives achieves TPR = 1, FPR = 900/9900 ≈ 0.091 (AUC ≈ 0.955), yet only Precision = 100/(100 + 900) = 0.10 (AP ≈ 0.10), starkly revealing poor early retrieval performance.^56,57^ Furthermore, an individual coordinate in the ROC space does not uniquely specify a confusion matrix nor a set of matrices with an equivalent MCC,^58,59^ thus raising additional concerns about the reliability of ROC-AUC for benchmarking SSL classification models. In contrast, the MCC accounts for all four quadrants of the confusion matrix (i.e., sensitivity, specificity, precision, and negative predictive value), thus, requiring the classifier to excel across multiple dimensions in order to achieve a high MCC score. Indeed, while a high MCC value (e.g., 0.9) is invariably associated with a high ROC-AUC, the reverse does not necessarily hold.^60^ The discrepancy between these metrics is evident in Figure 2e, where the MCC for KGG remains below 50% on certain data sets, whereas its ROC-AUC values consistently surpass 60% (Figure 2a). For imbalanced binary classification tasks, the average precision (AP) metric further complements MCC by jointly considering recall and precision.^56^ Recognizing the inherent limitations of ROC-AUC and the need for more robust performance measures, we benchmarked KGG using both MCC and AP (Figure 2e). This approach represents the first instance of an SSL framework advocating MCC and AP as primary metrics for molecular classification, thereby offering a more comprehensive and reliable assessment of classification performance. Although the adoption of new metrics can introduce reproducibility challenges in large-scale benchmarking, we encourage future studies to consider MCC and AP alongside ROC-AUC to ensure robust and meaningful evaluations. To facilitate future comparative analyses, we present the MCC and AP performance of KGG in Table S3, thereby encouraging the broader adoption of more robust and informative evaluation metrics in molecular classification tasks.

## 3 Conclusions

In this study, we propose an extended graph representation that explicitly incorporates orbital information, demonstrating superior performance over conventional approaches across a range of chemical tasks within an SSL framework. By embedding orbital characteristics into molecular graphs, our proposed methodology effectively captures detailed structural and chemical nuances, thereby surpassing conventional state-of-the-art SSL techniques relying solely on one-hot encodings. The principal contributions of this research are (1) introducing two chemically informed knowledge vectors — hybridization states and bond types, to enrich molecular representations; (2) designing a novel pretext task focused on reconstructing the orbital information encapsulated in these vectors; and (3) conducting a data contamination analysis to evaluate the generalizability and robustness of the proposed model.

The key advantages of KGG are its data efficiency and architectural versatility, rooted in its orbital-informed motif-level representations. By distilling fundamental chemical heuristics from moderately sized corpora, it bypasses the need for exhaustive pretraining and excels in low-data fine-tuning scenarios. This robust, chemically grounded foundation serves as a potent front-end module, broadly enhancing diverse GNN architectures. Additionally, such chemically principled representations are crucial in materials science, where descriptors encoding local atomic environments and bonding topologies are vital for predicting formation energies, developing interatomic potentials, and designing novel catalysts.^27,61^ Whether the specific orbital-centric inductive bias of KGG offers a distinct advantage for predicting the emergent properties of extended solid-state systems remains a compelling avenue for future research.

Despite the very encouraging performance of KGG in molecular property prediction, certain limitations warrant further exploration. First, the framework currently lacks an analysis of model uncertainty, which future studies should incorporate to quantify the prediction confidence more effectively. Second, although chemical knowledge was utilized to encode select features, critical descriptors such as atom types remain represented via one-hot encoding, yielding high-dimensional feature spaces prone to overfitting. Addressing this challenge, subsequent research should focus on systematically transforming chemical domain knowledge (such as periodic groupings and periods, lone-pair contributions in conjugated systems, electron dynamics, and electron-density distributions) into graph-structured representations, thereby enhancing both interpretability and predictive performance. Third, scaling KGG to million-molecule corpora and unfamiliar chemistries will strain memory, computation, and generalization; hierarchical/multiscale graph architectures, memory-efficient subgraph batching, domain-adaptive fine-tuning, and noise-robust objectives are promising remedies. Fourth, pretraining tasks are pivotal for the success of downstream applications. Accordingly, the selection of samples in the pretraining data set must be designed not only to capture the intrinsic diversity of the data but also to prevent the introduction of bias or contamination, which can undermine generalization. Curating a pretraining corpus that maximizes structural diversity while minimizing scaffold bias will further bolster transfer to new chemical spaces.

Future work will generalize the KGG framework to tackle tasks beyond the molecular properties. A focus on reaction rather than substances could be supported by orbital-aware representations that extend graph representation of reactions, in particular the *Imaginary Transition State* graph or *Condensed graph of Reaction*.^62^ In parallel, we aim to enhance the framework’s interpretability by developing explainable GNN models.^63^ The goal is not only to decode the learned *Structure–Activity Relationships* for rational compound design but ultimately to visualize and understand the fundamental electron-pushing mechanisms that drive chemical transformations.

## 4 Methods

The overall framework of KGG is depicted in Figure 1 and comprises two distinct training stages: (1) pretraining with KSSP and (2) fine-tuning with KRG. Both stages start with the same processing pipeline: (1) construction of a hierarchical graph (Section 4.1), (2) integration of atom-level knowledge vectors (Section 4.2), and (3) application of a representation extractor to encode the hierarchical graph into a feature vector (Section 4.3). The resulting representation is then employed during both the pretraining and fine-tuning phases.

### 4.1 Hierarchical Graph

#### 4.1.1 Hierarchical Structure

Given a SMILES string, a molecular graph *G* = (*V*, *E*) is constructed using RDKit.^64^ The vertex set *V* is defined as *V* = {*v*_1_, *v*_2_, ..., *v*_*n*_}, where *n* is the number of atoms in the molecule. The edge set *E* is given by a pair of atoms (*v*_*i*_, *v*_*j*_) such that *v*_*i*_ and *v*_*j*_ are connected by a chemical bond.

Subsequently, KGG identifies chemically meaningful substructures (motifs) as subgraphs *M*_*i*_^′^ = (*V*_*i*_^′^, *E*_*i*_^′^) of *G*. The motif decomposition makes use of BRICS, a system of drug-like chemical fragments,^65^ and an additional rule from HiMol.^33^ The decomposition will select a vertex set of *G* that matches the BRICS and HiMol rules. Complete details on the motif decomposition procedure are provided in Supporting Section 3.1 and Algorithm S1. Each motif *M*_*i*_ is associated with a motif node *v*_*M*_*i*__ ∈ *V*_*m*_. Motif-atom edges *E*_*m*_ link motif nodes *v*_*M*_*i*__ to their constituent atom nodes *v*_*j*_, i.e., we introduce the edge (*v*_*M*_*i*__, *v*_*j*_) whenever *v*_*j*_ ∈ *V*(*M*_*i*_) where *V*(*M*_*i*_) is the set of atoms forming the motif subgraph *M*_*i*_.

To incorporate global structural information, a *graph-level node* or *supernode v*_*g*_ ∈*V*_*g*_ is introduced. Thus, the augmented hierarchical graph G̃ consists of three layers (*atom level*, *motif level*, *graph level*) and is formally represented as *G̃* = (*Ṽ*, *Ẽ*) with *Ṽ* = *V* ∪ *V*_*m*_ ∪ *V*_*g*_ and *Ẽ* = *E* ∪ *E*_*m*_ ∪ *E*_*g*_. The edges of *E*_*g*_ connect the supernode to all motif nodes. Figure 7a illustrates the hierarchical graph construction process.

**Figure 7:**
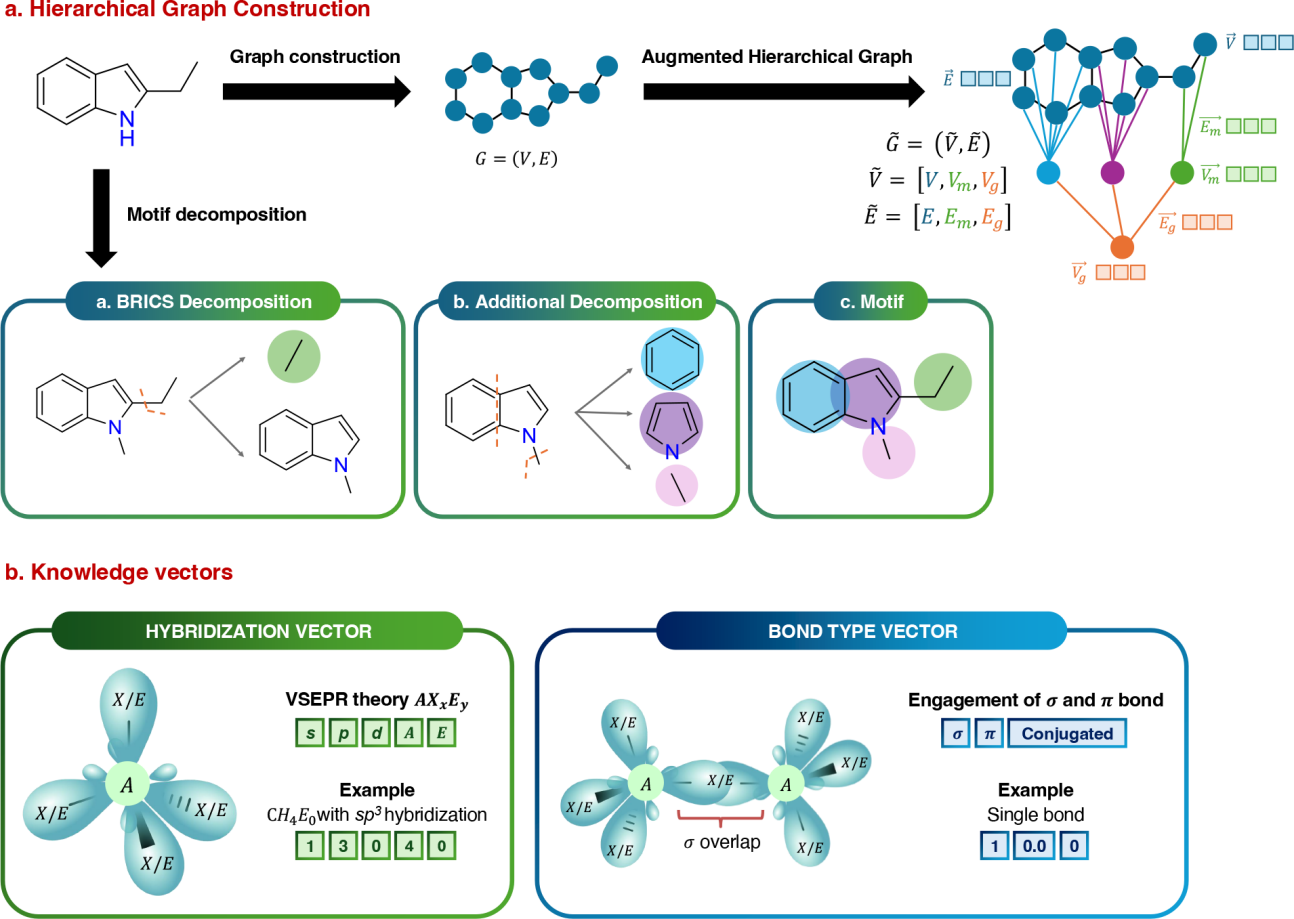
Molecular representation using KGG. (a) The three distinct levels of encoding. (b) The knowledge vector concept applied to atoms and bonds.

#### 4.1.2 Hierarchical Encoding

Let *V*, *V*_*m*_, and *V*_*g*_ denote the sets of atoms, motifs, and the supernode, respectively. The *full representation H* ^0^ of the hierarchical graph is defined as the concatenation of the atom-level, motif-level, and graph-level feature representations: *H* ^0^ = *H*∥*H*_*m*_∥*H*_*g*_, where ∥ denotes the vertical concatenation of matrices and vectors. The individual feature representations, using a *d*-dimensional embedding space, are defined as follows: $H=\left\{h_{v}|v\in V\right\}\subseteq R^{\left|V\right|\times d}$ is the atom-level feature matrix, where each atom feature vector **h**_*v*_ encodes *atom-type* and *bond-type* information, such as atomic number, hybridization state, and bond orders.

$H_{m}=\left\{h_{M_{i}}|M_{i}\in V_{m}\right\}\subseteq R^{\left|V_{m}\right|\times d}$ is the motif-level feature matrix. Here, each motif feature vector **h**_M_*i*__ captures the structural and chemical properties of motif *M*_*i*_. $H_{g}=h_{g}\in R^{d}$ is the graph-level feature vector **h**_*g*_, which aggregates global information from all motifs in *G̃*.

We choose a common embedding dimension *d* for atom-, motif- and graph-level features so that $H\in R^{\left|V\right|\times d}$ , $H_{m}\in R^{\left|V_{m}\right|\times d}$ , and $H_{g}\in R^{1\times d}$ , yielding the input tensor $H^{{}^{\circ}}=H∥H_{m}∥H_{g}\in R^{\left(|V|+|V_{m}|+1\right)\times d}$ , which (i) permits this concatenation as the encoder input, (ii) supports sentinel-based initialization (e.g., **h**_M_*i*__ = [120,0,···,0], **h**_*g*_ = [119,0,···,0]) to preserve level identity, and (iii) enables full weight sharing $W\in R^{d\times d}$ and cross-level comparisons via a single bilinear form without extra projections; e.g. for benzene (|*V*|= 6, |*V*_*m*_|= 1) with *d* = 7, we stack a 6 × 7 atom matrix, a 1 × 7 motif row, and a 1 × 7 graph row into an 8 × 7 tensor.

### 4.2 Knowledge Vectors

All feature descriptors are computed entirely algorithmically from the molecular graph (Supporting Section 3.2.1). We employ RDKit^64^ exclusively for SMILES parsing and to extract basic atom and bond properties; all higher-level orbital and bond descriptors are generated by our proprietary routines.

#### 4.2.1 Node Representation

The input features of atoms are atomic number, degree, and hybridization, as described in Supporting Section 3.2.2. However, conventional one-hot encodings often overlook the inherent relationships among the distinct hybridization states. In response, we use a knowledge vector-based encoding scheme that enrich molecular representations by incorporating both hybrid orbital compositions and features from VSEPR theory,^66^ as summarized in Figure 7b.

Let H denote the set of possible hybridization states for a given atom:

$$H=\left\{\mathrm{UNSPECIFIED},s,\mathrm{sp},sp^{2},sp^{3},sp^{3}d,sp^{3}d^{2}\right\}$$

We define the *hybridization function* $\phi_{\mathrm{hybrid}}:H\to N^{3}$ as the function that maps each state to (*n*_*s*_, *n*_*p*_, *n*_*d*_), where *n*_*s*_, *n*_*p*_, and *n*_*d*_ denote the numbers of *s*, *p*, and *d* orbitals.

VSEPR theory correlates the molecular shape with the arrangement of electron pairs around a central atom. The VSEPR descriptors, denoted AX_*x*_E_*y*_, can be formally defined by the *VSEPR function*: $\phi_{\mathrm{VSEPR}}:V\to N^{2}$ that maps a molecular geometry to (*x*, *y*), where *x* is the number of atoms *X* bonded to the central atom *A* and *y* is the number of lone pairs *E* on *A*.

To integrate hybridization with VSEPR descriptors into a single structural representation, we define the *composite function*: $\phi_{\mathrm{comp}}:H\times V\to N^{5}$ by ϕ_comp_(state, geometry) = (*n*_*s*_, *n*_*p*_, *n*_*d*_, *x*, *y*), where (*n*_*s*_, *n*_*p*_, *n*_*d*_) = ϕ_hybrid_(state) and (*x*, *y*) = ϕ_VSEPR_(geometry) .

As an example, consider the carbon atom in methane CH_4_E_0_, illustrated in Figure 7b. Using our scheme, we represent this as ϕ_comp_(C) = (1, 3, 0, 4, 0) . Here, 1 corresponds to the *s* orbital, 3 to the *p* orbitals, 0 to the absent of *d* orbitals, 4 indicates the total number of *sp* ^3^ orbitals, and 0 indicates no lone pairs on the carbon atom.

#### 4.2.2 Edges Representation

The initial bond features comprise the bond type, ring state, and knowledge vector that encodes distinct orbital contributions (see Supporting Section 3.2.3). This knowledge vector arises from quantum mechanical orbital interactions and is given by

$${\mathrm{e⃗}}_{ij}=\left(\begin{array}{c} \sigma_{ij}\\ \pi_{ij}\\ \delta_{ij} \end{array}\right)$$

where σ_*ij*_, π_*ij*_, and δ_*ij*_ represent the sigma, pi, and conjugation contributions, respectively, each determined by the relevant orbital overlap integrals. Figure 7b provides a visual representation of these bond contributions. For example, the single bond in ethane, Figure 7b (right), is represented as *e⃗*_*ij*_ = (1,0.0, 0)^T^ where 1 denotes the single σ-bond contribution, 0.0 indicates no π-bond component, and 0 denotes the absence of any conjugated state.

### 4.3 Molecular Representation Extractor

The objective of this stage is to encode the molecular graph features of an individual molecule into a 1D vector for training purposes.

Specifically, consider the hierarchical graph *G̃* = (*Ṽ*, *Ẽ*), where the node set is defined as *Ṽ* = *V* ∪*V*_*m*_ ∪*V*_*g*_ and the edge set as *Ẽ* = *E* ∪*E*_*m*_ ∪*E*_*g*_. The initial feature associated with a node *v*_*i*_ ∈ *Ṽ* is given by

$$X_{v_{i}}\in R^{d_{v}}$$

while the feature corresponding to an edge *e*_*vi*_ *v*_*j*_ ∈ *Ẽ* is denoted by

$$X_{v_{i}v_{j}}\in R^{d_{e}}$$

Here, *X*_*v*_*i*__ is a 7-dimensional feature vector assigned to node *v*_*i*_, and *X*_*v*_*i*_ *v*_*j*__ is a 5-dimensional feature vector characterizing the connection between nodes *v*_*i*_ and *v*_*j*_. An example of these feature vectors is illustrated in Figure 8 to enhance interpretability.

**Figure 8:**
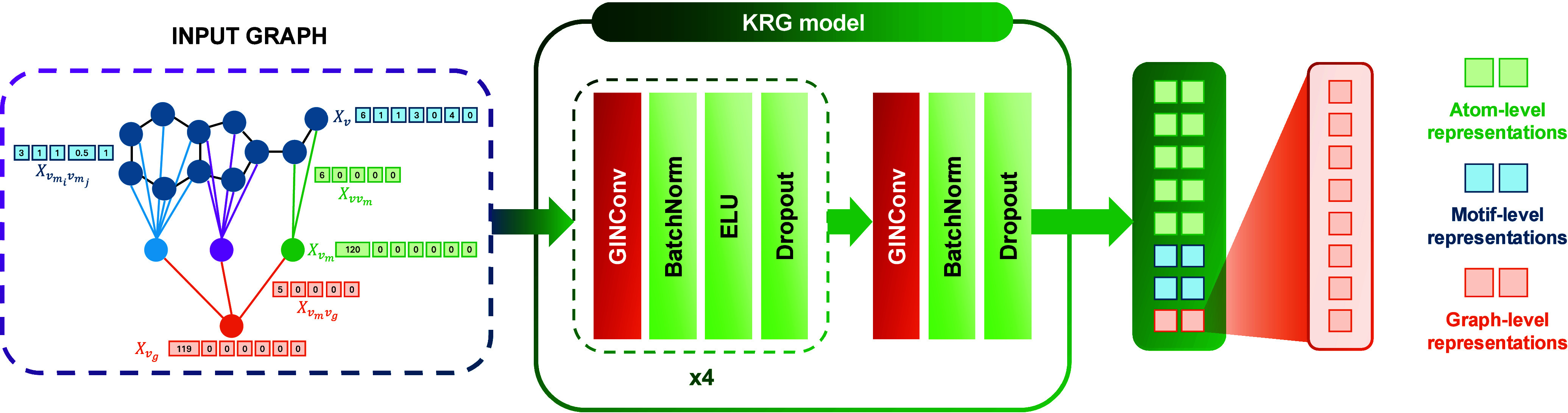
An overview of the KRG encoder, which consists of five GINConv layers.

These feature vectors are then transformed by an MLP prior to their use in the GINConv layer, as expressed in

$$h_{v_{i}}^{0}=\overset{d_{v}}{\underset{k=1}{\sum }}{\mathrm{MLP}}_{k}\left(x_{v_{i}}^{k}\right)$$

$$h_{v_{i}v_{j}}^{0}=\overset{d_{e}}{\underset{k=1}{\sum }}{\mathrm{MLP}}_{k}\left(x_{v_{i}v_{j}}^{k}\right)$$

where $x_{v}^{k}\in R$ is the *k*-th scalar component of the node feature vector *X*_*v*_*i*__ and $x_{v_{i}v_{j}}^{k}\in R$ is the *k*-th scalar component of the edge feature vector *X*_*v*_*i*_ *v*_*j*__. Here, *h*_*v*_*i*__^0^ and *h*_*v*_*i*_ *v*_*j*__^0^ denote the input feature embeddings for node *v*_*i*_ and edge *v*_*i*_ *v*_*j*_, respectively.

Next, *h*_*v*_*i*__^0^ and *h*_*v*_*i*_ *v*_*j*__^0^ are passed through a GINConv layer, defined by eq 1:

1 $$h_{v_{i}}^{\left(l\right)}={\mathrm{MLP}}^{\left(l\right)}\left(h_{v_{i}}^{\left(l-1\right)}+\underset{v_{j}\in N\left(v_{i}\right)}{\sum }\left(h_{v_{j}}^{\left(l-1\right)}+h_{v_{i}v_{j}}^{0}\right)\right)$$

where MLP ^(*l*)^ = {*Linear*(*d*, 2*d*) →ReLU →Linear­(2*d*, *d*)}, *h*_*v*_*i*__^(*l*–1)^ and *h*_*v*_*j*__^(*l*–1)^ are the embedding vectors of atoms *v*_*i*_ and *v*_*j*_ at layer *l* – 1, *h*_*v*_*i*__^(*l*)^ denotes the embedding vector of atom *v*_*i*_ at layer *l*, and N(v) denotes the set of neighboring nodes of *v*_*i*_ in the graph. For brevity, we write eq 1 as

$$h_{v_{i}}^{\left(l\right)}=\mathrm{GINConv}\left(h_{v_{i}}^{\left(l-1\right)},h_{v_{i}v_{j}}^{0}\right)$$

The KRG encoding model comprises five GINConv layers (Figure 8), interspersed with BatchNorm and Dropout layers. The feature update process for node *v*_*i*_ from the first to the fourth GINConv layer is given by

$$h_{v_{i}}^{\left(l\right)}={\mathrm{Dropout}}^{\left(l\right)}\left(\mathrm{ELU}\left({\mathrm{BatchNorm}}^{\left(l\right)}\left({\mathrm{GINConv}}^{\left(l\right)}\left(h_{v_{i}}^{\left(l-1\right)},h_{v_{i}v_{j}}^{0}\right)\right)\right)\right)$$

while, in the final and fifth GINConv layer, the ELU activation function is omitted:

$$h_{v_{i}}^{\left(5\right)}={\mathrm{Dropout}}^{\left(5\right)}\left({\mathrm{BatchNorm}}^{\left(5\right)}\left({\mathrm{GINConv}}^{\left(5\right)}\left(h_{v_{i}}^{\left(4\right)},h_{v_{i}v_{j}}^{0}\right)\right)\right)$$

Inspired by the success of Graphormer^67^ and Himol,^33^ a READOUT function is not employed to obtain the global graph representations. Instead, we adopt the supernode embedding *h*_*v*_*g*__^(5)^ (indicated in orange in Figure 8) as the representation of the entire graph. This embedding is subsequently used for both the pretext and downstream tasks. Further details of the dimensional transformation from the KRG encoder’s inputs to its outputs can be found in Supporting Section 3.3.

### 4.4 Pretrained Model

The pretraining KSSP model is derived from 11 pretext tasks (Figure 9) and the configurations for this stage is detailed in Supporting Section 3.6.1. Eight of these tasks focus on reconstructing two knowledge vectors, one task addresses the reconstruction of adjacency matrices, and two tasks target the prediction of graph-level properties. A key aspect of this pretraining scheme lies in reconstructing orbital information embedded in two knowledge vectors (hybridization and bond types), which play a pivotal role in defining chemical semantics yet have been overlooked in prior studies.^33–36,40,41,46–52^ By requiring the encoder to reconstruct a compact “knowledge vector” of orbital-inspired signals from the bare molecular graph, we guide its latent space to align with chemically meaningful concepts, such as bond strength, polarity, and reactive sites, that downstream predictors can readily exploit.

**Figure 9:**
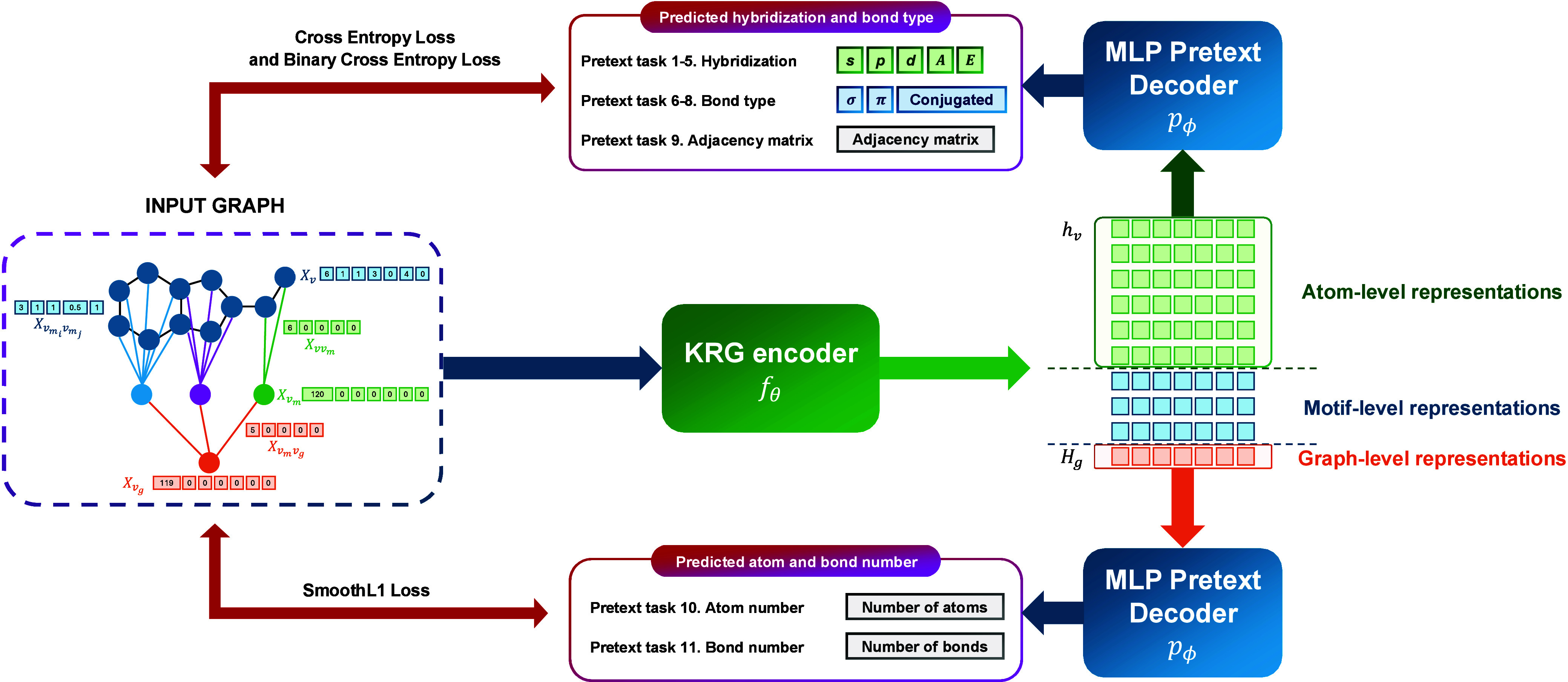
An architecture of the KSSP decoder.

#### Reconstruction of Hybridization Vector

We utilize *atomic-level* embeddings (*h*_*v*_*i*__) and employ Cross Entropy Loss (CELoss) to reconstruct five elements of ϕ_comp_(state, geometry) = (*n*_*s*_, *n*_*p*_, *n*_*d*_, *x*, *y*) as follows:

$$\begin{array}{c} h_{v_{i}}\to \phi_{n_{s}}\left\{\mathrm{Linear}\left(d,d\right)\to \mathrm{ReLU}\to \mathrm{Linear}\left(d,2\right)\right\}\to {ŷ}_{s}\\ h_{v_{i}}\to \phi_{n_{p}}\left\{\mathrm{Linear}\left(d,d\right)\to \mathrm{ReLU}\to \mathrm{Linear}\left(d,3\right)\right\}\to {ŷ}_{p}\\ h_{v_{i}}\to \phi_{n_{d}}\left\{\mathrm{Linear}\left(d,d\right)\to \mathrm{ReLU}\to \mathrm{Linear}\left(d,4\right)\right\}\to {ŷ}_{d}\\ h_{v_{i}}\to \phi_{x}\left\{\mathrm{Linear}\left(d,d\right)\to \mathrm{ReLU}\to \mathrm{Linear}\left(d,7\right)\right\}\to {ŷ}_{x}\\ h_{v_{i}}\to \phi_{y}\left\{\mathrm{Linear}\left(d,d\right)\to \mathrm{ReLU}\to \mathrm{Linear}\left(d,7\right)\right\}\to {ŷ}_{y} \end{array}$$

The hybridization loss is defined as the aggregate of five individual loss components, as detailed in eq 2:

2 $$L_{\mathrm{hybridization}}=L_{n_{s}}^{\mathrm{CELoss}}+L_{n_{p}}^{\mathrm{CELoss}}+L_{n_{d}}^{\mathrm{CELoss}}+L_{x}^{\mathrm{CELoss}}+L_{y}^{\mathrm{CELoss}}$$

#### Reconstruction of Bond Type Vector

Bond formation arises from axial (σ) and lateral (π) orbital overlaps, which determine properties such as the bond strength and length. Consequently, predicting these overlaps enables the model to capture essential chemical information. The three bond-type tasks are defined as follows:

$$\begin{array}{c} \mathrm{concat}\left[h_{v_{i}},h_{v_{j}}\right]\to \phi_{\sigma }\left\{\mathrm{Linear}\left(2d,d\right)\to \mathrm{ReLU}\to \mathrm{Linear}\left(d,1\right)\right\}\to {ŷ}_{\sigma }\\ \mathrm{concat}\left[h_{v_{i}},h_{v_{j}}\right]\to \phi_{\pi }\left\{\mathrm{Linear}\left(2d,d\right)\to \mathrm{ReLU}\to \mathrm{Linear}\left(d,1\right)\right\}\to {ŷ}_{\pi }\\ \mathrm{concat}\left[h_{v_{i}},h_{v_{j}}\right]\to \phi_{\mathrm{conjugation}}\left\{\mathrm{Linear}\left(2d,d\right)\to \mathrm{ReLU}\to \mathrm{Linear}\left(d,1\right)\right\}\to {ŷ}_{\mathrm{conjugation}} \end{array}$$

Here, *h*_*v*_*i*__ and *h*_*v*_*j*__ are the atomic-level embeddings of the two atoms *v*_*i*_ and *v*_*j*_ forming a covalent bond. The total bond type loss is the sum of three individual losses:

$$L_{\mathrm{bond}\text{\_}\mathrm{type}}=L_{\sigma }^{\mathrm{BCE}}+L_{\pi }^{\mathrm{SmoothL}1}+L_{\mathrm{conjugation}}^{\mathrm{BCE}}$$

#### Adjacency Matrix Reconstruction

This task is defined by

$$\mathrm{concat}\left[h_{v_{i}},h_{v_{j}}\right]\to \phi_{\mathrm{adj}}\left\{\mathrm{Linear}\left(2d,d\right)\to \mathrm{ReLU}\to \mathrm{Linear}\left(d,1\right)\right\}\to {ŷ}_{v_{i}v_{j}}$$

where *y*_*v*_*i*_ *v*_*j*__ ∈{0,1} indicates whether a bond exists between atoms *v*_*i*_ and *v*_*j*_. The loss for adjacency matrix reconstruction is

$$L_{\mathrm{adj}}=-\underset{v_{i},v_{j}\in V}{\sum }\left(y_{v_{i}v_{j}}\log{ŷ}_{v_{i}v_{j}}+\left(1-y_{v_{i}v_{j}}\right)\log\left(1-{ŷ}_{v_{i}v_{j}}\right)\right)$$

#### Graph-Level Prediction Tasks

We utilized the *graph-level* embedding (*h*_*g*_) to predict the number of atoms and the number of bonds. These tasks are expressed as

$$\begin{array}{c} h_{g}\to \phi_{\mathrm{atoms}}\left\{\mathrm{Linear}\left(d,\frac{d}{4}\right)\to \mathrm{Softplus}\to \mathrm{Linear}\left(\frac{d}{4},1\right)\right\}\to {ŷ}_{\mathrm{atoms}}\\ h_{g}\to \phi_{\mathrm{bonds}}\left\{\mathrm{Linear}\left(d,\frac{d}{4}\right)\to \mathrm{Softplus}\to \mathrm{Linear}\left(\frac{d}{4},1\right)\right\}\to {ŷ}_{\mathrm{bonds}} \end{array}$$

Here, *ŷ*_atoms_ and *ŷ*_bonds_ are the predicted values for the number of atoms and bonds, respectively. The loss function SmoothL1Loss is less sensitive to outliers compared to MSE^68^ and can according to Girshick^69^ help prevent gradient explosions. Based on SmoothL1Loss, we formulate the losses as

$$\begin{array}{c} L_{\mathrm{atoms}}=\{\begin{array}{cc} \frac{1}{2}{∥y_{\mathrm{atoms}}-{ŷ}_{\mathrm{atoms}}∥}_{2}^{2}, & \mathrm{if}{∥y_{\mathrm{atoms}}-{ŷ}_{\mathrm{atoms}}∥}_{1}<1\\ {∥y_{\mathrm{atoms}}-{ŷ}_{\mathrm{atoms}}∥}_{1}-\frac{1}{2}, & \mathrm{otherwise} \end{array}\\ L_{\mathrm{bonds}}=\{\begin{array}{cc} \frac{1}{2}∥y_{\mathrm{bonds}}-{ŷ}_{\mathrm{bonds}}{∥}_{2}^{2}, & \mathrm{if}∥y_{\mathrm{bonds}}-{ŷ}_{\mathrm{bonds}}{∥}_{1}<1\\ {∥y_{\mathrm{bonds}}-{ŷ}_{\mathrm{bonds}}∥}_{1}-\frac{1}{2}, & \mathrm{otherwise} \end{array} \end{array}$$

#### Objective Loss Function

Finally, the total loss for back-propagation is the sum of all five principal losses, as represented in eq 3:

3 $$L=L_{\mathrm{hybridization}}+L_{\mathrm{bond}\text{\_}\mathrm{type}}+L_{\mathrm{adj}}+L_{\mathrm{atoms}}+L_{\mathrm{bonds}}$$

### 4.5 Data Sets and Baselines

For the pretraining stage, approximately 250,000 molecules were randomly sampled from the ZINC15 data set.^70^ The model was fine-tuned using 12 molecular property data sets from MoleculeNet,^53^ comprising six classification and six regression tasks. Further details on both the pretraining and fine-tuning data sets are provided in the Supporting Section 2.1 and summarized in Table S13. Following established studies,^33–36,40,41,46–52^ we adopted a *scaffold* splitting strategy to divide each data set into training, validation, and test subsets at an 8:1:1 ratio, thereby ensuring structural differences between training and test molecules and assessing model generalizability. Subsequently, we benchmarked the KGG model against a set of state-of-the-art self-supervised learning baselines. Further details regarding these benchmark methods are available in Supporting Section 2.2.

### 4.6 Experimental Settings

#### Training Configurations

The research was conducted on a Linux System 22.04 LTS, powered by an IntelCorei7-13700K processor featuring 16 processing units and 24 CPUs operating at 3.10 GHz. The system includes 512 GB of memory and 96 GB of DDR4 RAM, with graphics capabilities provided by a GTX 4070 Ti Super card containing 16 GB of VRAM.

During both pretraining and fine-tuning phases, the Adam optimizer was utilized, along with a batch size of 32 and an embedding dimensionality of 512. Fine-tuning was repeated three times per data set with distinct random seeds. Further training configuration details can be found in Supporting Section 3.6.

#### Comparisons with Traditional Fingerprints

To comprehensively assess the molecular representation capabilities of the KGG neural graph fingerprints—specifically, global representations extracted from the fine-tuned KRG layers of the KGG model—were generated for two classification data sets (BACE and BBBP) and four regression data sets (ESOL, FreeSolv, Lipophilicity, and QM7). Subsequently, these embeddings were evaluated using an kNN classifier with *n*

textsubscript*n*eighbors = 3 to quantify classification performance through the ROC-AUC metric, as well as to measure predictive accuracy on regression tasks using RMSE and MAE. For comparative purposes, identical analyses were performed with three established molecular fingerprints: MACCS,^71^ ECFP4,^72^ and RDK7.^73^ Data set partitioning was conducted according to the strategy described in Section 4.5. Additionally, the discriminative power of the KGG fingerprints was further illustrated through visualization of the embeddings using the t-SNE^74^ algorithm, clearly demonstrating their capacity to effectively distinguish molecular structures within a two-dimensional embedding space.

#### Data Contamination

The methodology for the data contamination assessment comprises two principal steps: (1) extracting every compound from the test sets of each fine-tuning data set and (2) evaluating the extent of overlap between these compounds and those in the pretraining data set, with equality tests based on normalized canonical SMILES structures. This normalization and comparison is conducted using the RDKit library to ensure consistency and accuracy in detecting potential data contamination.

## Supplementary Material



## Data Availability

All code and the pretrained KGG model are publicly available at https://github.com/ThinhUMP/KGGraph. The ZINC data set employed for pretraining is downloadable from https://github.com/ZangXuan/HiMol, as outlined in Himol.^33^ Furthermore, the downstream data sets used for fine-tuning can be obtained through the MoleculeNet repository at https://github.com/deepchem/deepchem/tree/master/deepchem/molnet/load_function.

## Notes

Additional experimental procedures, data sets, benchmarks, and supplementary figures and tables (PDF)
