# Supplementary material for: KGG: Knowledge-Guided Graph Self-Supervised Learning to Enhance Molecular Property Predictions
Source: J Chem Inf Model. 2025 Sep 8;65(18):9443–58. doi: 10.1021/acs.jcim.5c01068 (PMC12458709; doi:10.1021/acs.jcim.5c01068)
Supplement: Supplementary file 1 [file ci5c01068_si_001.pdf]

Supporting Information

KGG: Knowledge-Guided Graph Self-Supervised Learning to Enhance Molecular Property Predictions

Van-Thinh To,[†] Phuoc-Chung Van Nguyen,[†] Gia-Bao Truong,[†] Tuyet-Minh Phan,[†] Tieu-Long Phan,[*,‡,¶] Rolf Fagerberg,[¶] Peter F. Stadler,[‡,§,∥,⊥,#,@] and Tuyen Ngoc Truong[*,†]

†*Faculty of Pharmacy, University of Medicine and Pharmacy at Ho Chi Minh City, 41 Dinh Tien Hoang, District 1, Ho Chi Minh City, 700000, Vietnam.*

‡*Bioinformatics Group, Department of Computer Science & Interdisciplinary Center for Bioinformatics & School for Embedded and Composite Artificial Intelligence (SECAI), Leipzig University, Härtelstraße 16–18, D-04107 Leipzig, Germany*

¶*Department of Mathematics and Computer Science, University of Southern Denmark, DK-5230 Odense M, Denmark*

§*Max Planck Institute for Mathematics in the Sciences, Inselstraße 22, D-04103, Leipzig, Germany*

∥*Department of Theoretical Chemistry, University of Vienna, Währingerstraße 17, A-1090, Vienna, Austria*

⊥*Facultad de Ciencias, Universidad National de Colombia,Bogotá, Colombia*

#*Center for non-coding RNA in Technology and Health, University of Copenhagen,*

Ridebanevej 9, DK-1870, Frederiksberg, Denmark

@*Santa Fe Institute, 1399 Hyde Park Rd., Santa Fe, NM, 87501, USA*

E-mail: tieu@bioinf.uni-leipzig.de; truongtuyen@ump.edu.vn

1 Additional Figures and Tables

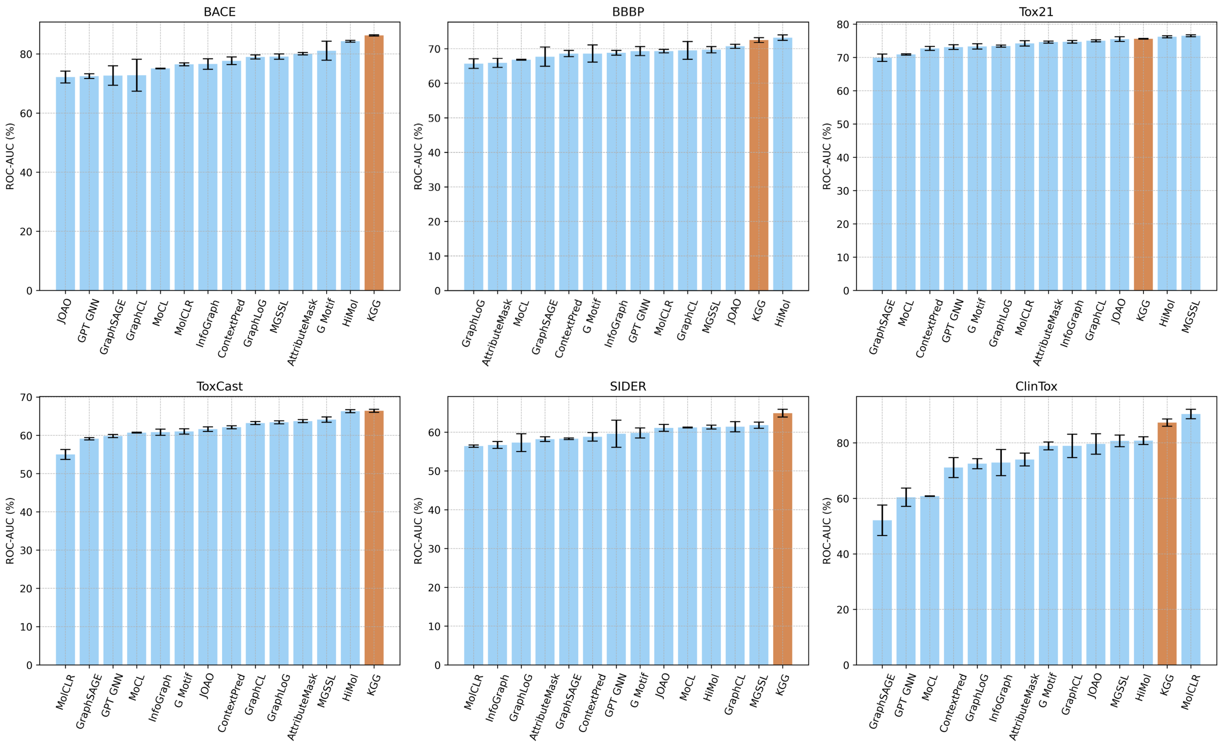

Figure S1: ROC-AUC Results and Standard Deviations for Classification Datasets.

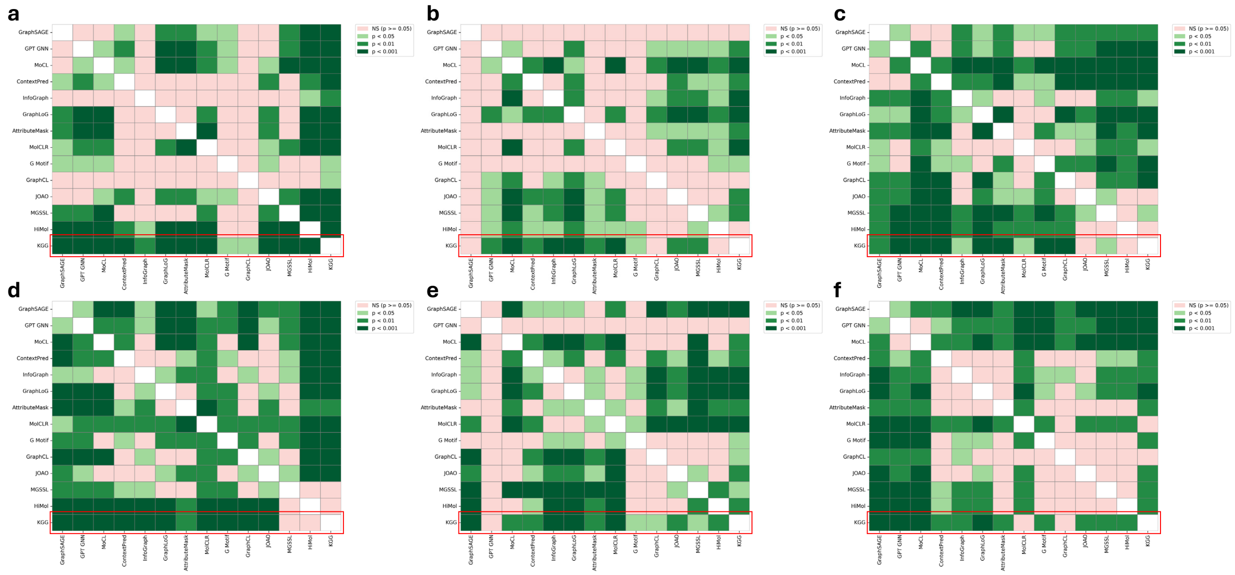

Figure S2: T-test Statistical Analysis for Classification Datasets. **(a)** BACE. **(b)** BBBP. **(c)** Tox21. **(d)** ToxCast. **(e)** SIDER. **(f)** ClinTox.

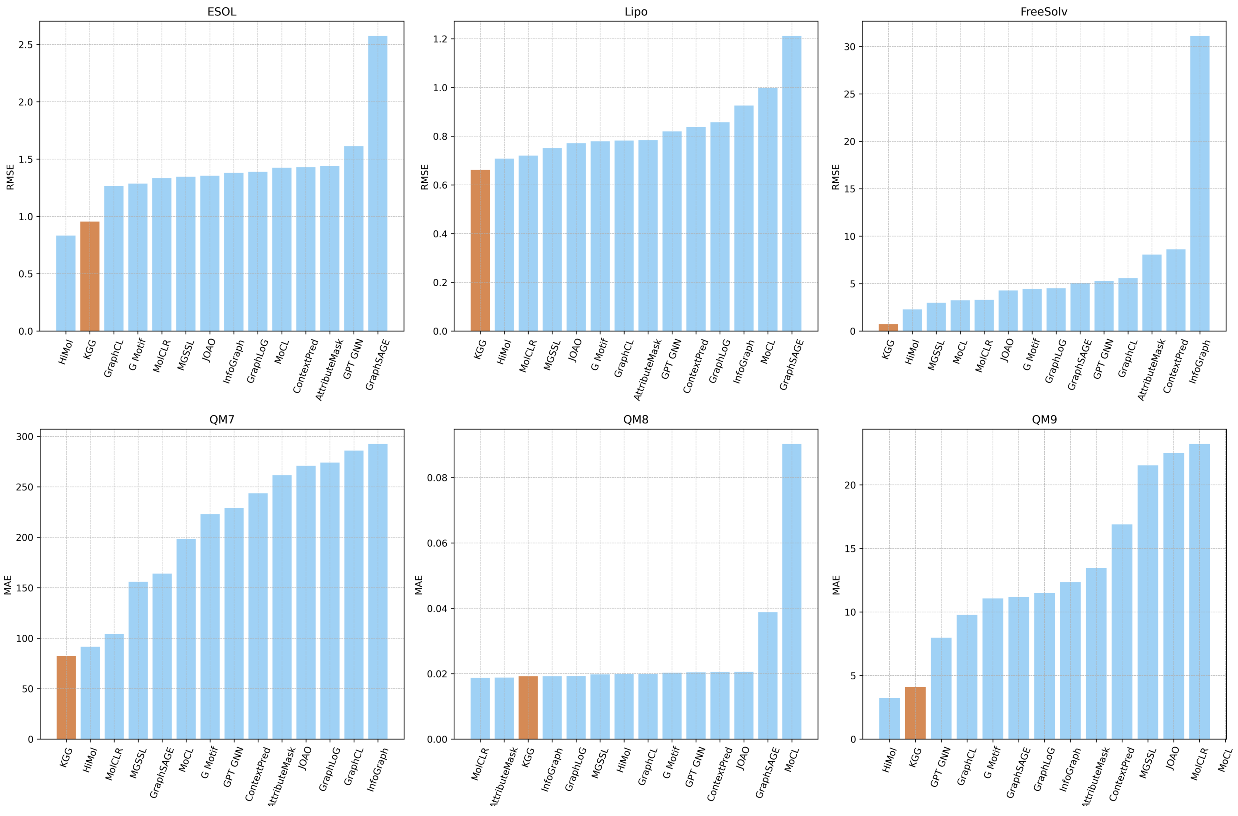

Figure S3: Comparison of `SSL` Models on Regression Datasets: RMSE and MAE Metrics.

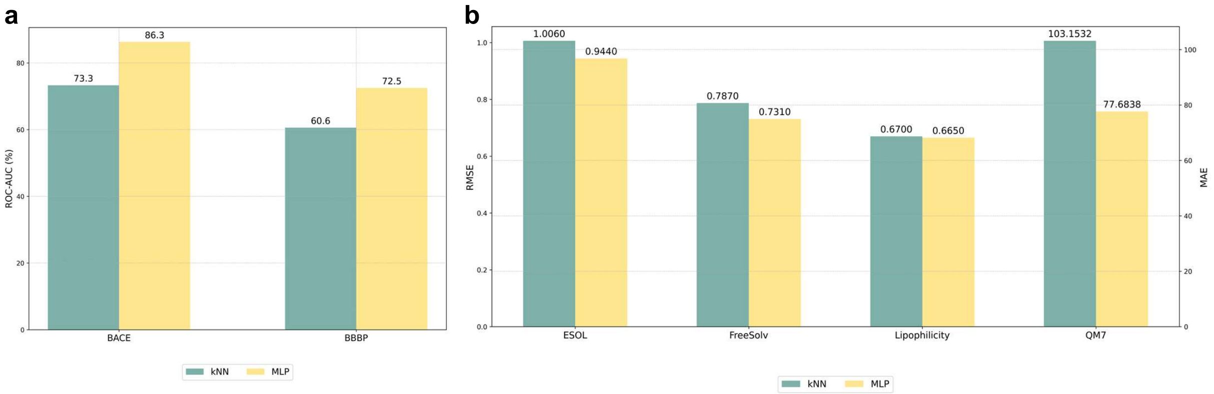

Figure S4: **(a)** Comparison of `kNN` and `MLP` performance using the `KGG` synthetic graph fingerprint for the BACE and BBBP datasets. **(b)** Comparison of `kNN` and `MLP` performance using the `KGG` synthetic graph fingerprint for the four regression datasets ESOL, FreeSolv, Lipophilicity, and QM7.

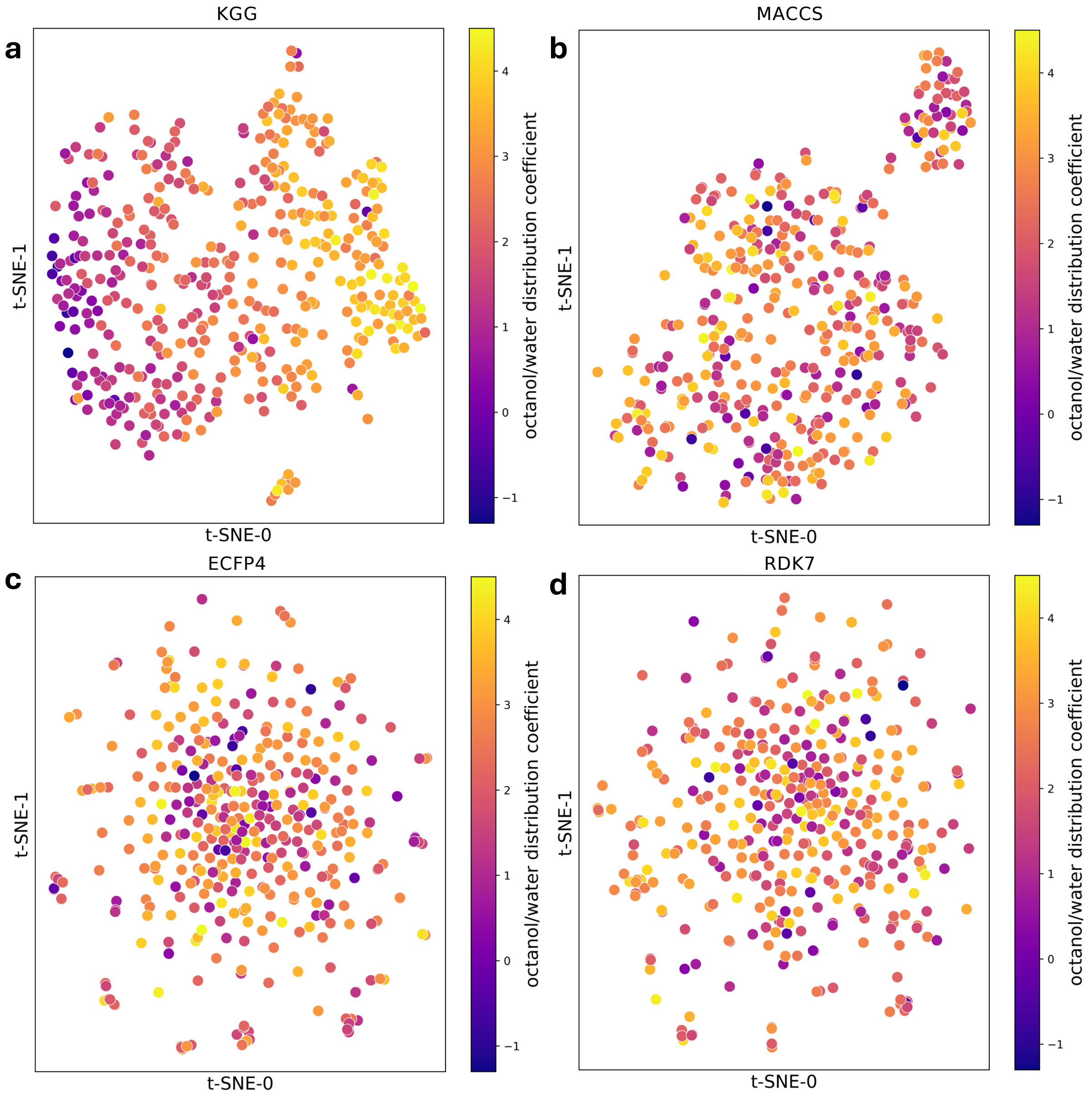

Figure S5: t-SNE visualization of the test set for the Lipophilicity dataset using four types of fingerprints: **(a)** KGG, **(b)** MACCS, **(c)** ECFP4, and **(d)** RDK7.

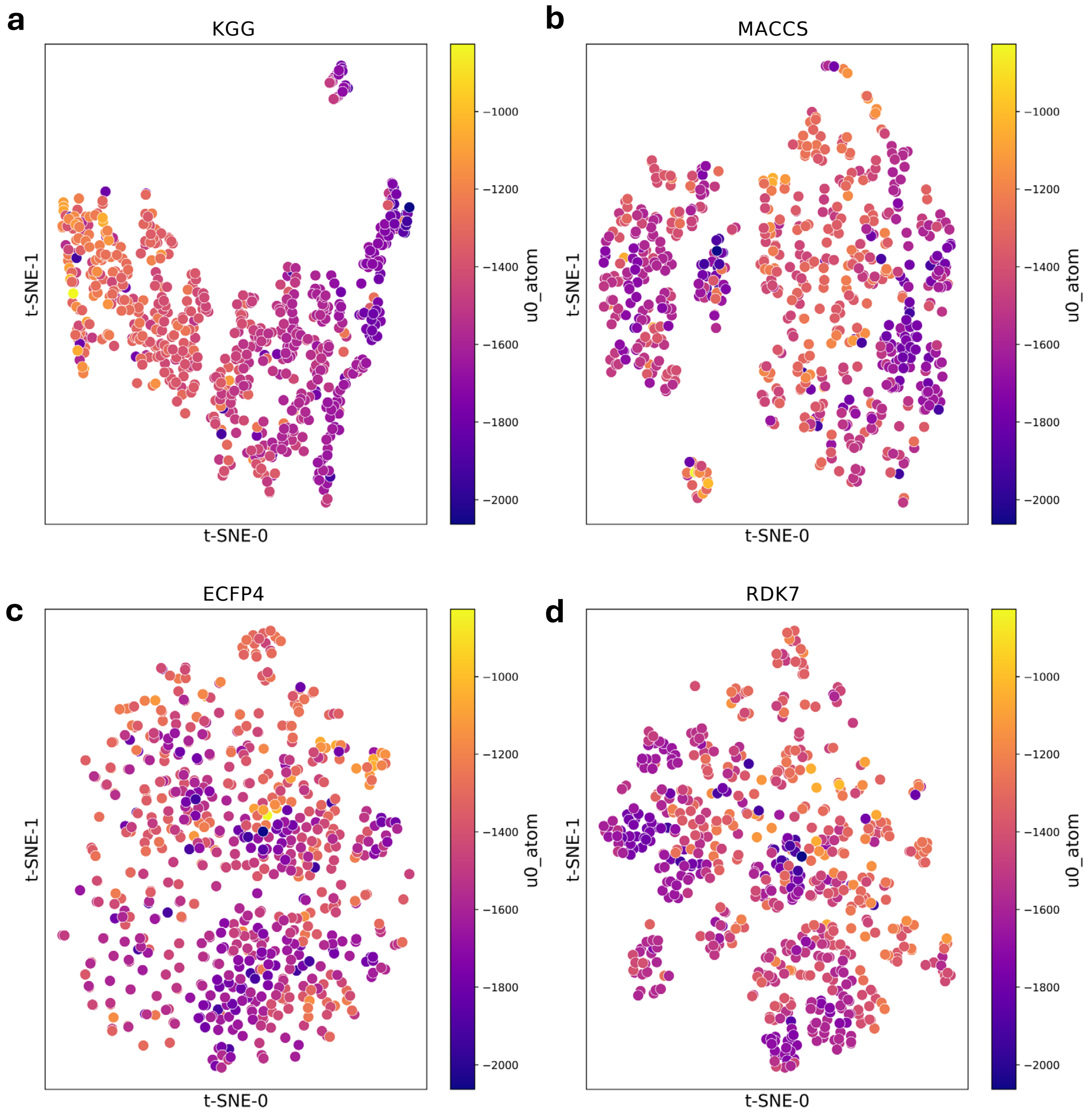

Figure S6: `t-SNE` visualization of the test set for the QM7 dataset using four types of fingerprints: **(a)** `KGG`, **(b)** `MACCS`, **(c)** `ECFP4`, and **(d)** `RDK7`.

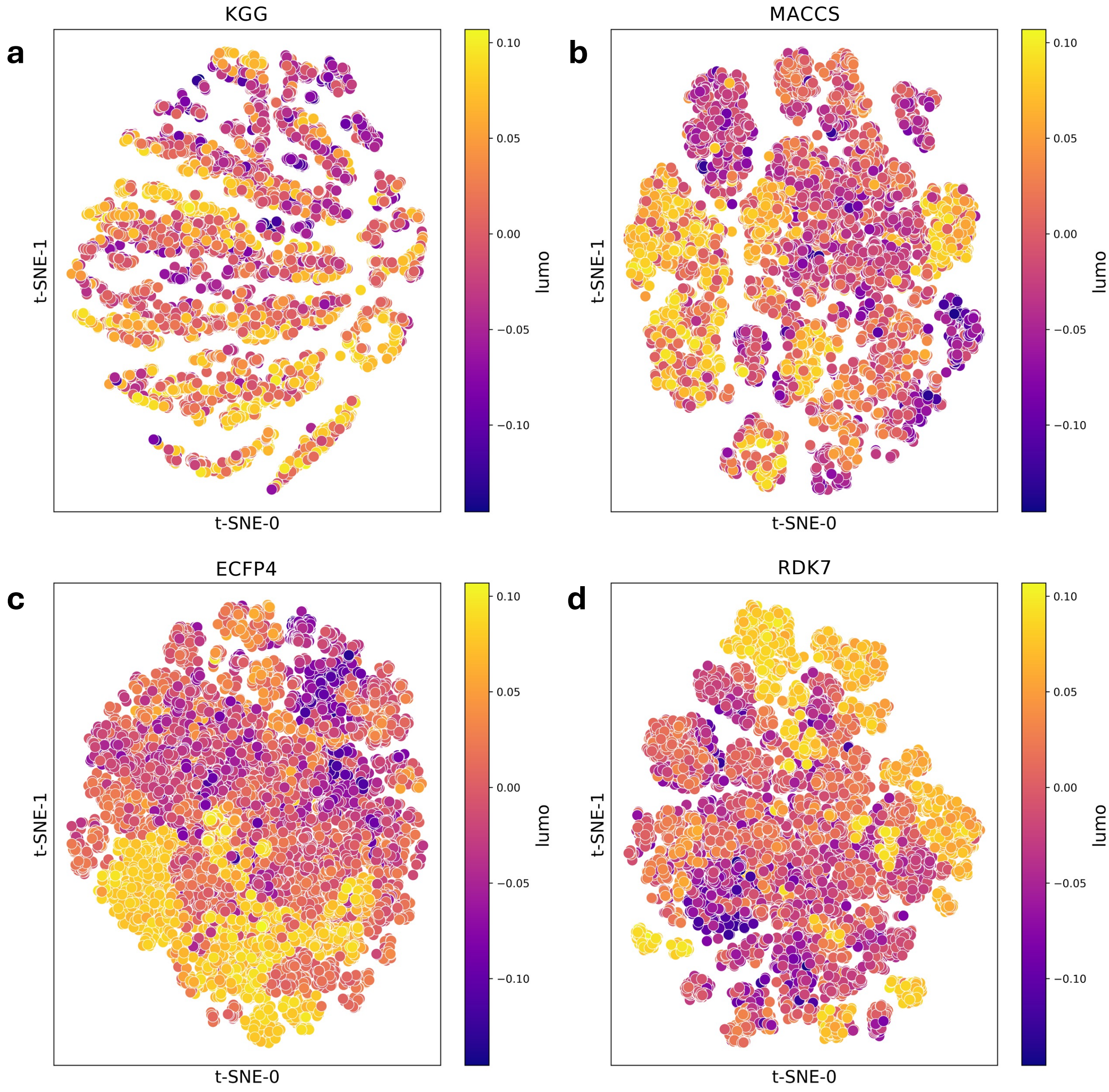

Figure S7: `t-SNE` visualization of the test set for the QM9 dataset using four types of fingerprints: **(a)** `KGG`, **(b)** `MACCS`, **(c)** `ECFP4`, and **(d)** `RDK7`.

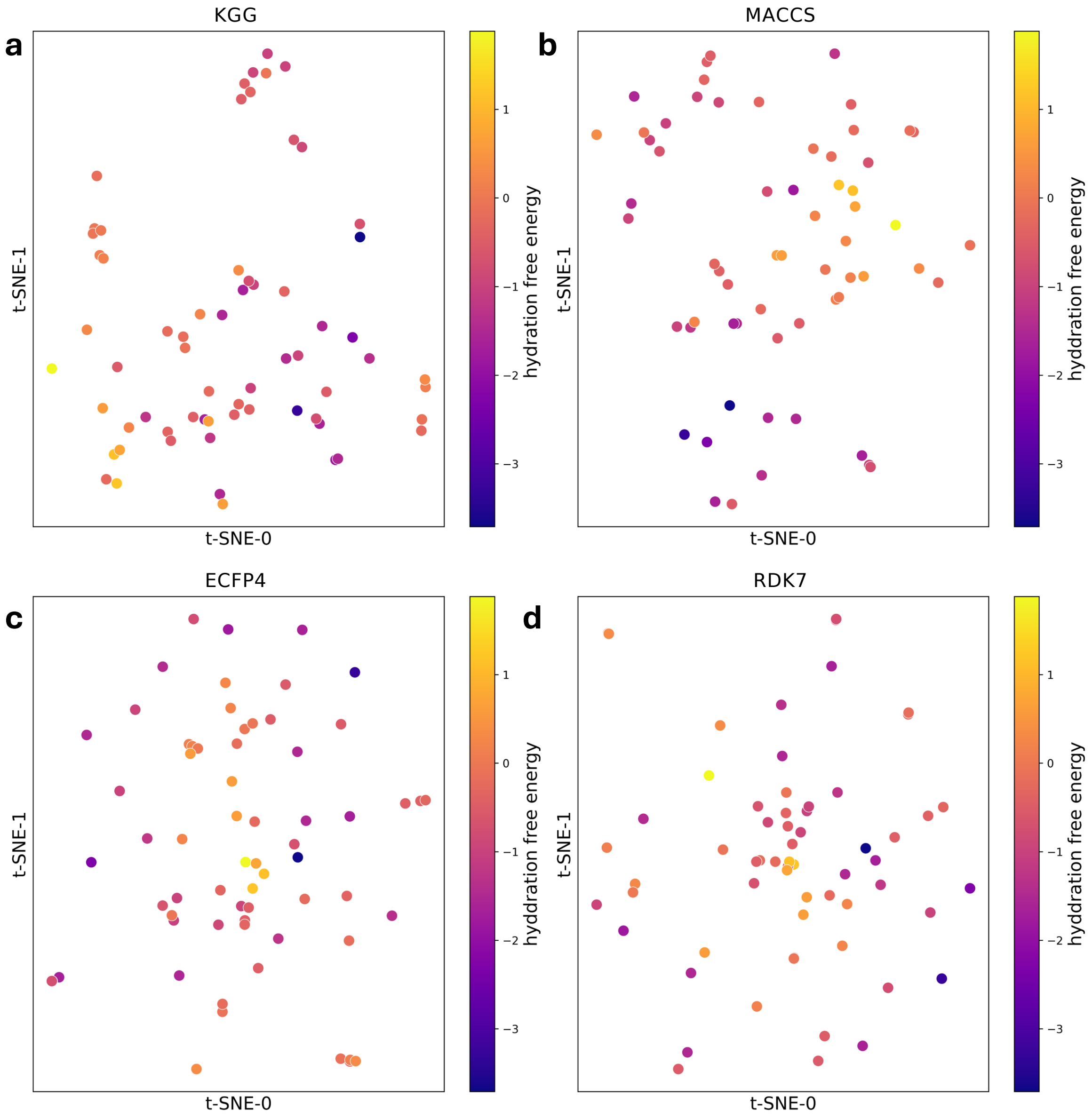

Figure S8: `t-SNE` visualization of the test set for the FreeSolv dataset using four types of fingerprints: **(a)** `KGG`, **(b)** `MACCS`, **(c)** `ECFP4`, and **(d)** `RDK7`.

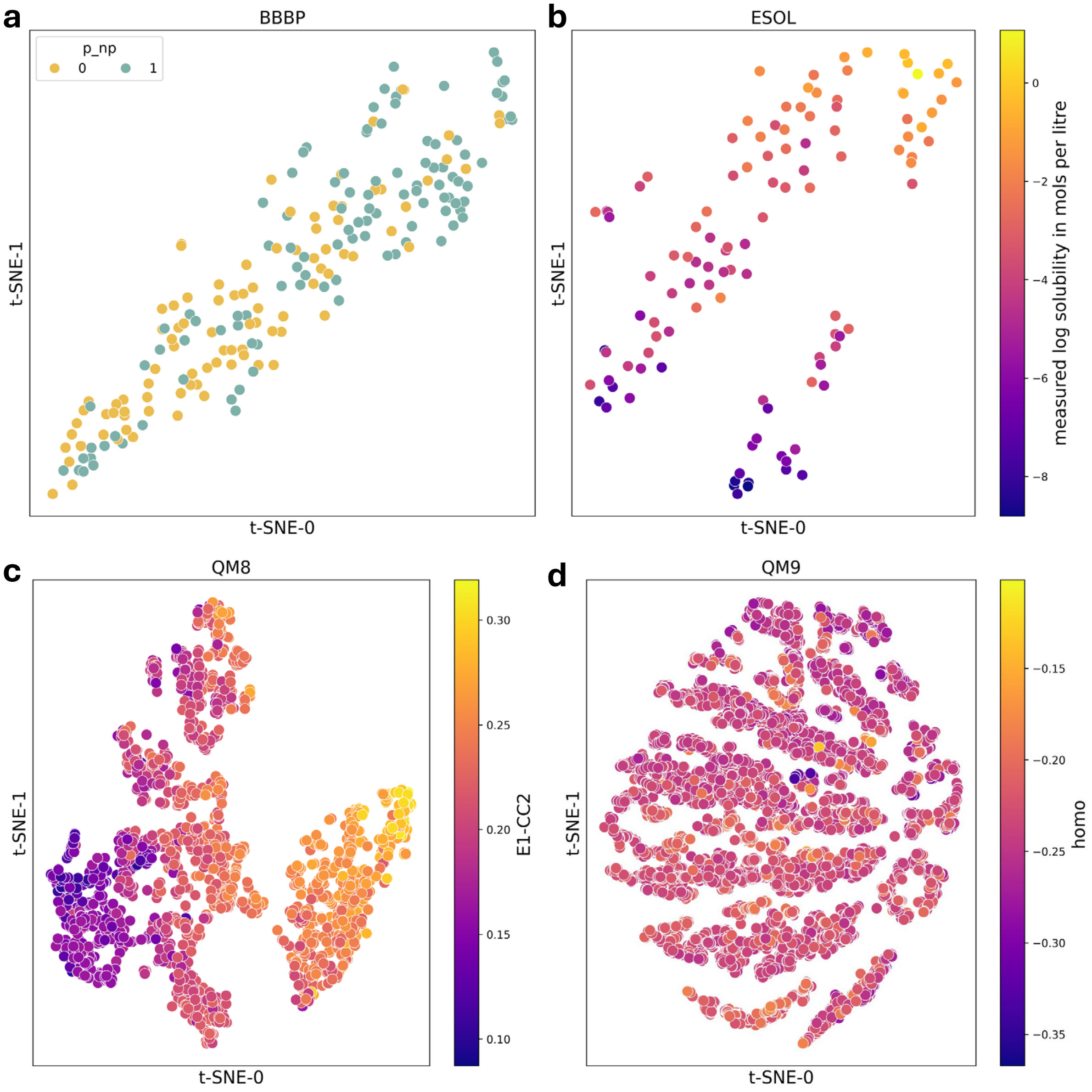

Figure S9: `t-SNE` visualization of the test set of four datasets using `KGG` fingerprints: **(a)** BBBP, **(b)** ESOL, **(c)** QM8, and **(d)** QM9.

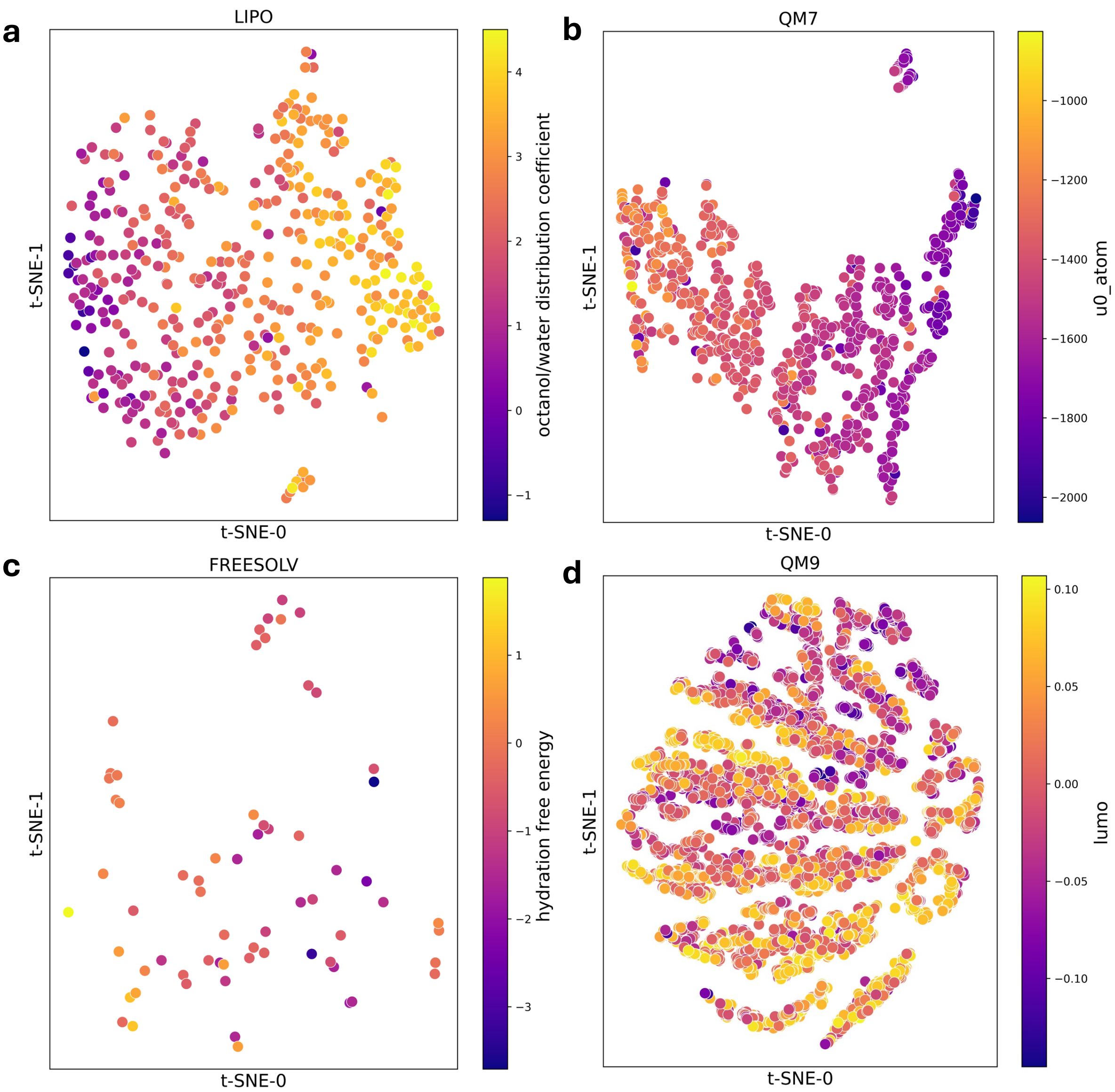

Figure S10: `t-SNE` visualization of the test set of four datasets using `KGG` fingerprints: **(a)** Lipo (octanol/water distribution coefficient), **(b)** QM7 (u0_atom), **(c)** FreeSolv (hydration free energy), and **(d)** QM9 (lumo).

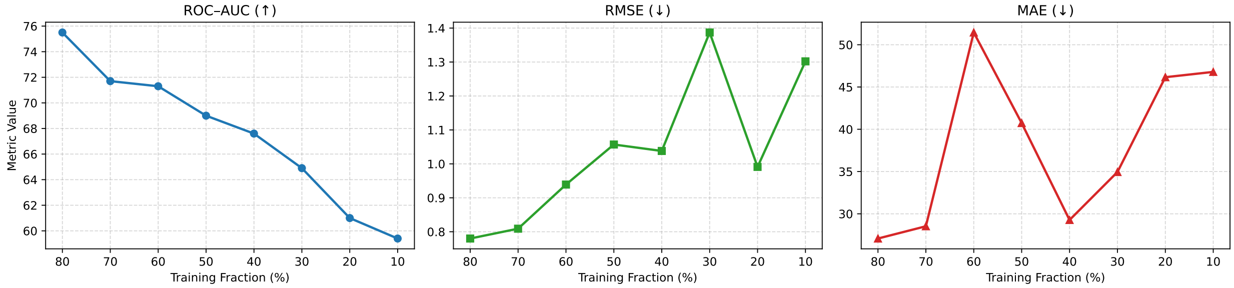

Figure S11: Effect of training set size on `KGG` performance.

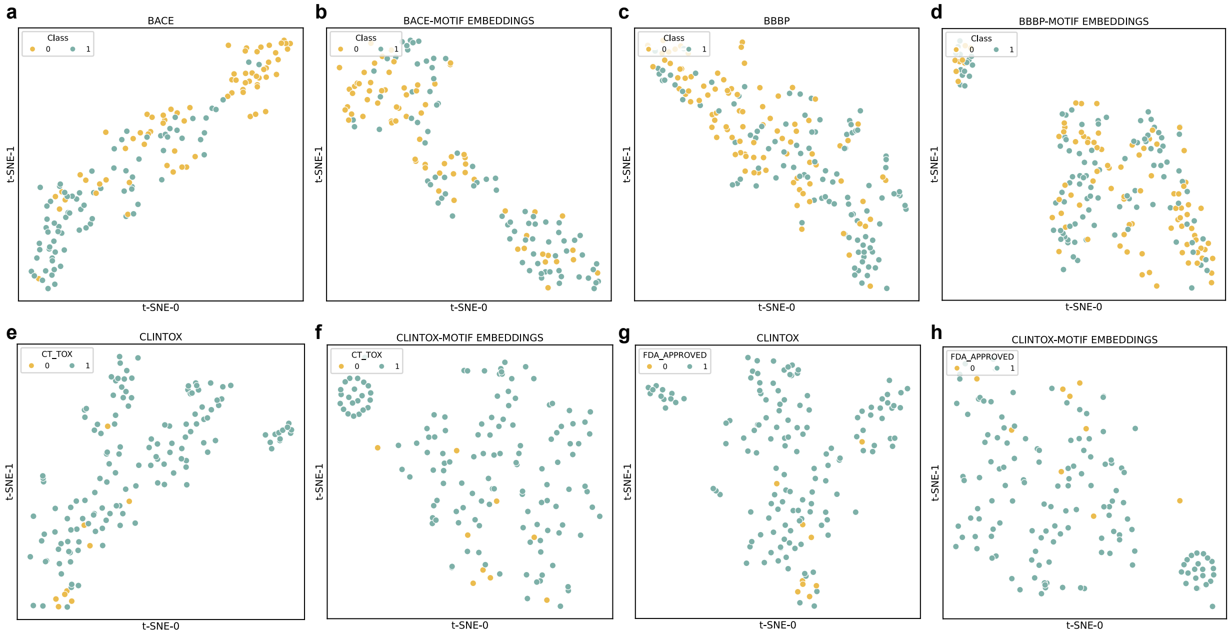

Figure S12: Two–dimensional `t-SNE` comparison between *graph-level* embeddings (subplots **a**, **c**, **e**, **g**) and the corresponding aggregated motif embeddings (subplots **b**, **d**, **f**, **h**) on the test splits of three classification benchmarks. Each point represents a molecule and is color-coded by its ground-truth class label. Dataset–specific subplots: BACE (**a**,**b**), BBBP (**c**,**d**), and ClinTox (**e**-**h**).

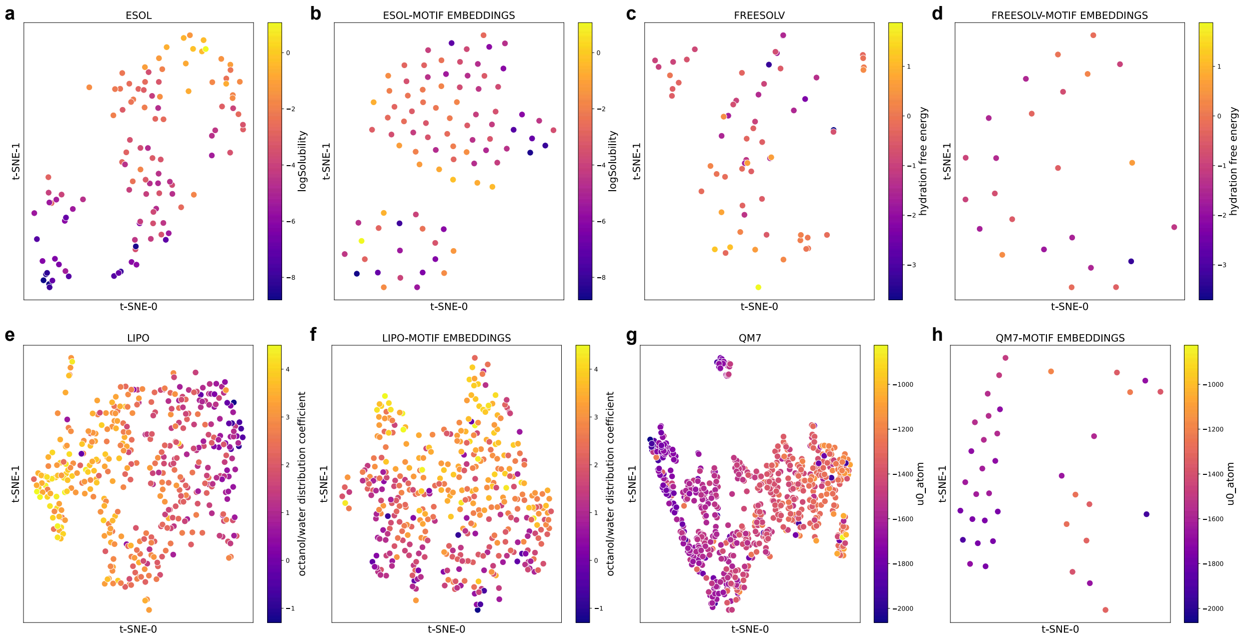

Figure S13: Two–dimensional `t-SNE` comparison between *graph-level* embeddings (subplots **a**, **c**, **e**, **g**) and the corresponding aggregated motif embeddings (subplots **b**, **d**, **f**, **h**) on the test splits of four regression benchmarks. Each point represents a molecule and is color-coded by its ground-truth class label. Dataset–specific subplots: ESOL (**a**,**b**), FreeSolv (**c**,**d**), Lipophilicity (**e**,**f**), and QM7 (**g**, **h**).

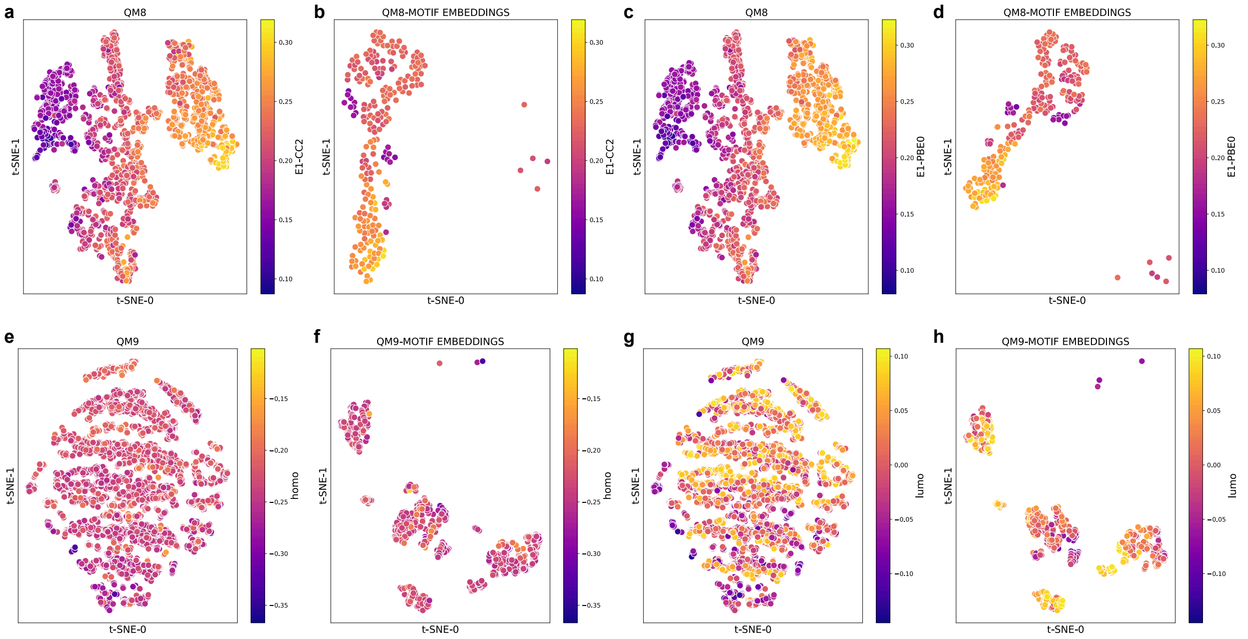

Figure S14: Two–dimensional `t-SNE` comparison between *graph-level* embeddings (subplots **a**, **c**, **e**, **g**) and the corresponding aggregated motif embeddings (subplots **b**, **d**, **f**, **h**) on the test splits of two regression benchmarks. Each point represents a molecule and is color-coded by its ground-truth class label. Dataset–specific subplots: QM8 (**a-d**), and QM9 (**e-h**).

Table S1: Performance comparison on molecular classification benchmarks from MoleculeNet. Results are reported as the mean and standard deviation of ROC-AUC (%), evaluated across six binary classification tasks. Each value is computed from three independent experiments using different random seeds. The **bold** values indicate the best performance, and underlined values indicate the second-best performance. The symbol (↑) denotes that higher values indicate better performance.

**Model**	**BACE**	**BBBP**	**Tox21**	**ToxCast**	**SIDER**	**ClinTox**	**Avg.* (↑)**
GraphSAGE	72.7 ± 3.3	67.7 ± 2.8	69.9 ± 1.1	59.1 ± 0.3	58.3 ± 0.2	52.1 ± 5.5	63.3
GPT_GNN	72.5 ± 0.8	69.3 ± 1.3	73.1 ± 0.7	59.8 ± 0.4	59.6 ± 3.5	60.4 ± 3.3	65.8
AttributeMask	80.1 ± 0.4	65.9 ± 1.3	74.6 ± 0.3	63.7 ± 0.4	58.2 ± 0.6	74.0 ± 2.3	69.4
ContextPred	77.7 ± 1.3	68.6 ± 0.9	72.7 ± 0.6	62.1 ± 0.4	58.8 ± 1.1	71.1 ± 3.6	67.7
InfoGraph	76.6 ± 1.8	68.8 ± 0.7	74.7 ± 0.4	60.8 ± 0.8	56.7 ± 0.9	72.9 ± 4.7	68.4
MoCL	75.1 ± 0.1	66.8 ± 0.1	70.9 ± 0.2	60.7 ± 0.1	61.2 ± 0.1	60.8 ± 0.1	65.9
GraphLoG	79.0 ± 0.7	65.7 ± 1.4	73.4 ± 0.3	63.4 ± 0.4	57.3 ± 2.3	72.5 ± 1.8	68.6
GraphCL	72.8 ± 5.4	69.5 ± 2.6	75.0 ± 0.3	63.2 ± 0.4	61.4 ± 1.3	78.9 ± 4.2	71.5
JOAO	72.2 ± 2.0	70.7 ± 0.6	75.5 ± 0.7	61.6 ± 0.6	61.1 ± 0.9	79.6 ± 3.7	71.8
MolCLR	76.5 ± 0.5	69.3 ± 0.5	74.2 ± 0.8	55.0 ± 1.3	56.4 ± 0.3	**90.4 ± 1.7**	70.3
G_Motif	81.1 ± 3.2	68.6 ± 2.5	73.3 ± 0.8	61.0 ± 0.7	59.8 ± 1.3	78.9 ± 1.4	70.5
MGSSL	79.1 ± 0.9	69.7 ± 0.9	**76.5 ± 0.3**	64.1 ± 0.7	61.8 ± 0.8	80.7 ± 2.1	72.0
HiMol	84.3 ± 0.3	**73.2 ± 0.8**	76.2 ± 0.3	66.3 ± 0.4	61.3 ± 0.5	80.8 ± 1.4	73.7
KGG (ours)	**86.3 ± 0.2**	72.5 ± 0.7	75.6 ± 0.1	**66.4 ± 0.4**	**64.9 ± 1.0**	87.3 ± 1.3	**75.5**

**Average ROC-AUC (%) across six datasets: BACE, BBBP, Tox21, ToxCast, SIDER, ClinTox.*

Table S2: Comparative performance of models on molecular property regression tasks from the MoleculeNet datasets. The table reports both root mean square error (RMSE) for ESOL, FreeSolv, and Lipophilicity (Lipo) tasks, and mean absolute error (MAE) for the QM7, QM8, and QM9 tasks. The **bold** values indicate the best performance, and underlined values indicate the second-best performance. The symbol (↓) denotes that lower values indicate better performance.

Model	ESOL RMSE	FreeSolv RMSE	Lipo RMSE	Avg.* RMSE (↓)	QM7 MAE	QM8 MAE	QM9 MAE	Avg.** MAE (↓)
GraphSAGE	2.575	5.051	1.212	2.946	164.062	0.0388	11.178	58.426
GPT_GNN	1.612	5.284	0.820	2.572	229.053	0.0204	7.976	79.016
AttributeMask	1.439	8.062	0.784	3.428	261.588	0.0188	13.461	91.689
ContextPred	1.430	8.616	0.838	3.628	243.551	0.0205	16.886	86.819
InfoGraph	1.380	31.118	0.926	11.141	292.601	0.0192	12.350	101.657
MoCL	1.425	3.233	0.998	1.885	198.215	0.0903	NA	99.153
GraphLoG	1.390	4.515	0.857	2.254	274.071	0.0193	11.484	95.191
GraphCL	1.265	5.569	0.782	2.539	285.967	0.0199	9.773	98.587
JOAO	1.355	4.280	0.771	2.135	270.839	0.0206	22.507	97.789
MolCLR	1.333	3.285	0.720	1.779	104.184	**0.0187**	23.226	42.476
G_Motif	1.286	4.432	0.779	2.166	222.957	0.0203	11.065	78.014
MGSSL	1.346	2.980	0.751	1.692	155.913	0.0198	21.538	59.157
HiMol	**0.833**	2.283	0.708	1.275	91.501	0.0199	**3.243**	31.588
KGG (ours)	0.944	**0.731**	**0.665**	**0.780**	**77.6838**	0.019	3.526	**27.076**

**Average RMSE across three datasets: ESOL, FreeSolv, Lipo. **Average MAE across three datasets: QM7, QM8, QM9.*

Table S3: Performance of KGG in terms of Average Precision (AP) (%) and Matthews Correlation Coefficient (MCC) (%). Results are reported as the mean and standard deviation of AP (%) and MCC (%), evaluated across six classification datasets from MoleculeNet. The symbol (↑) denotes that higher values indicate better performance.

	BACE	BBBP	Tox21	ToxCast	SIDER	ClinTox	Avg. (↑)
**AP (%)**	85.0 ± 0.3	74.3 ± 0.8	34.3 ± 0.8	34.8 ± 0.9	63.9 ± 1.0	65.6 ± 1.3	59.6
**MCC (%)**	48.7 ± 1.2	31.9 ± 0.5	27.3 ± 1.0	8.8 ± 0.4	12.7 ± 0.7	31.6 ± 1.1	26.8

Table S4: Ablation study of `KGG` variants for the pre-training process, as detailed in Supporting Section 3.4. The table reports ROC-AUC scores (%) for six classification datasets across different `KGG` pre-training configurations. The **bold** values indicate the best performance, and underlined values indicate the second-best performance. The symbol (↑) denotes that higher values indicate better performance.

**Model**	**BACE**	**BBBP**	**Tox21**	**ToxCast**	**SIDER**	**ClinTox**	**Avg. (↑)**
`KGGwoPre`	77.4	46.8	69.6	58.2	57.3	53.9	60.5
`KGGHyBowoPre`	80.8	69.4	75.4	63.4	60.0	77.5	<u>71.1</u>
`KGG`	86.3	72.5	75.6	66.4	64.9	87.3	**75.5**

Table S5: Ablation study of `KGG` model variants for different featurization approaches, as represented in Supporting Section 3.4. The table reports ROC-AUC scores (%) on six benchmark classification datasets. Each variant disables specific representations (such as hybridization and bond type vectors) to assess their contribution to overall performance. `KGG` denotes the full model with all components enabled. The **bold** values indicate the best performance, and underlined values indicate the second-best performance. The symbol (↑) denotes that higher values indicate better performance.

**Model**	**BACE**	**BBBP**	**Tox21**	**ToxCast**	**SIDER**	**ClinTox**	**Avg. (↑)**
`KGGwoHybri`	80.1	67.3	74.4	63.3	61.8	58.9	67.6
`KGGonehot`	84.0	67.1	73.6	62.8	61.3	61.1	68.3
`KGGwoHyBo`	81.6	68.1	73.1	62.2	61.2	66.3	68.8
`KGGwoBondType`	79.1	71.1	76.1	66.0	63.3	85.7	<u>73.6</u>
`KGG`	86.3	72.5	75.6	66.4	64.9	87.3	**75.5**

Table S6: ROC-AUC (%) of `KGG` with different `GNN` backbones (`GIN`, `GCN`, `GAT` and `GraphSAGE`) on six MoleculeNet classification benchmarks. The **bold** values indicate the best performance, and underlined values indicate the second-best performance. Higher values indicate better performance (↑).

**Backbone**	**BACE**	**BBBP**	**Tox21**	**ToxCast**	**SIDER**	**ClinTox**	**Avg.* (↑)**
`GIN`	86.3	72.5	75.6	66.4	64.9	87.3	**75.5**
`GCN`	80.8	64.8	74.9	65.1	63.0	90.3	<u>73.2</u>
`GAT`	73.3	56.3	68.6	58.7	53.9	46.6	59.6
`GraphSAGE`	81.8	67.6	76.9	65.7	61.2	83.0	72.7

**Average ROC-AUC (%) across six datasets: BACE, BBBP, Tox21, ToxCast, SIDER, ClinTox.*

Table S7: Performance of `KGG` with `GIN`, `GCN`, `GAT` and `GraphSAGE` backbones on MoleculeNet regression benchmarks. For ESOL, FreeSolv and Lipophilicity (Lipo) we report RMSE; for QM7, QM8 and QM9 we report MAE. The **bold** values indicate the best performance, and underlined values indicate the second-best performance. Lower values indicate better performance (↓).

**Backbone**	**ESOL** RMSE	**FreeSolv** RMSE	**Lipo** RMSE	**Avg.*** (↓) RMSE	**QM7** MAE	**QM8** MAE	**QM9** MAE	**Avg.**** (↓) MAE
`GIN`	0.944	0.731	0.665	**0.780**	77.684	0.019	3.562	<u>27.076</u>
`GCN`	0.925	0.682	0.797	<u>0.801</u>	78.203	0.022	4.357	27.527
`GAT`	1.510	0.701	1.016	1.076	130.555	0.024	64.521	65.033
`GraphSAGE`	0.997	0.728	0.845	0.857	71.439	0.021	4.363	**25.274**

**Avg.* (↓): average RMSE over ESOL, FreeSolv, Lipo. ***Avg.* (↓): average MAE over QM7, QM8, QM9.

Table S8: Performance of `KGG` as the training datasets are reduced. Classification tasks are reported with ROC-AUC (↑); regression tasks use RMSE or MAE (↓). The symbols (↑) and (↓) indicate that higher or lower values, respectively, reflect better performance.

**Task type**	**Dataset**	**Metric**	**80 %**	**70 %**	**60 %**	**50 %**	**40 %**	**30 %**	**20 %**	**10 %**
Classification	BACE	ROC–AUC ↑	86.3	82.7	79.3	78.9	77.6	77.0	74.5	62.9
	BBBP	ROC–AUC ↑	72.5	69.9	72.6	69.1	66.6	58.5	50.1	42.8
	Tox21	ROC–AUC ↑	75.6	73.4	71.9	67.2	70.1	58.8	51.4	65.4
	ToxCast	ROC–AUC ↑	66.4	61.3	59.2	59.6	55.9	57.1	55.3	56.0
	SIDER	ROC–AUC ↑	64.9	62.0	59.4	59.0	60.9	54.8	54.1	54.5
	Clintox	ROC–AUC ↑	87.3	80.9	85.4	80.1	74.5	83.3	80.5	74.6
	*Average**	ROC–AUC ↑	75.5	71.7	71.3	69.0	67.6	64.9	61.0	59.4
Regression	ESOL	RMSE ↓	0.944	0.998	1.288	1.057	1.539	2.264	1.327	1.908
	FreeSolv	RMSE ↓	0.731	0.687	0.715	0.786	0.662	0.881	0.911	0.921
	Lipo	RMSE ↓	0.665	0.741	0.815	0.799	0.913	1.017	1.070	1.076
	Average†	RMSE ↓	0.780	0.809	0.939	1.057	1.038	1.387	0.991	1.302
	QM7	MAE ↓	77.6838	80.0168	149.1791	116.1691	80.5083	96.5960	114.4575	113.4418
	QM8	MAE ↓	0.019	0.020	0.024	0.026	0.025	0.028	0.033	0.027
	QM9	MAE ↓	3.526	5.541	5.137	6.014	7.281	8.206	23.985	26.891
	Average‡	MAE ↓	27.076	28.526	51.447	40.736	29.271	34.943	46.159	46.787

* Average ROC-AUC (%) over six classification datasets: BACE, BBBP, Tox21, ToxCast, SIDER, and ClinTox. † Average RMSE over three regression datasets: ESOL, FreeSolv, and Lipophilicity. ‡ Average MAE over three quantum datasets: QM7, QM8, and QM9.

Table S9: Comparative performance of KGG on molecular classification benchmarks from the MoleculeNet under two distinct pre-training datasets. Results are reported in ROC-AUC (%), evaluated across six binary classification tasks. The symbol (↑) denotes that higher values indicate better performance.

**Model**	**BACE**	**BBBP**	**Tox21**	**ToxCast**	**SIDER**	**ClinTox**	**Avg.* (↑)**
ChEMBL	85.8	73.6	75.7	65.7	60.3	87.4	74.8
ZINC	86.3	72.5	75.6	66.4	64.9	87.3	75.5

**Average ROC-AUC (%) across six datasets: BACE, BBBP, Tox21, ToxCast, SIDER, ClinTox.*

Table S10: Comparative performance of KGG across molecular property regression benchmarks from the MoleculeNet, evaluated under two pre-training datasets. The table reports both root mean square error (RMSE) for ESOL, FreeSolv, and Lipophilicity (Lipo) tasks, and mean absolute error (MAE) for the QM7, QM8, and QM9 tasks. The symbol (↓) denotes that lower values indicate better performance.

**Model**	**ESOL** RMSE	**FreeSolv** RMSE	**Lipo** RMSE	**Avg.*** RMSE (↓)	**QM7** MAE	**QM8** MAE	**QM9** MAE	**Avg.**** MAE (↓)
ChEMBL	0.952	0.727	0.747	0.808	79.3990	0.019	3.710	27.709
ZINC	0.944	0.731	0.665	0.780	77.6838	0.019	3.526	27.076

**Average RMSE across three datasets: ESOL, FreeSolv, Lipo. **Average MAE across three datasets: QM7, QM8, QM9.*

Table S11: Data contamination analysis. For each SSL baseline we report the percentage of contamination. A lower contamination ratio indicates better generalization (↓).

**Model**	**Pretraining dataset**	**Molecules (approx.)**	**Cont. ratio (%) (↓) with QM datasets**	**Cont. ratio (%) (↓) without QM datasets**
GraphSAGE	NA	NA	NA*	NA*
GPT_GNN	NA	NA	NA*	NA*
AttributeMask	ZINC15	∼ 2 000 000	1.24	6.13
ContextPred	ZINC15	∼ 2 000 000	1.24	6.13
InfoGraph	QM9	∼ 134 000	82.66	1.39
MoCL	Downstream datasets	Downstream datasets	100.0	100
GraphLoG	ZINC15	∼ 2 000 000	1.24	6.13
GraphCL	ZINC15	∼ 2 000 000	1.24	6.13
JOAO	ZINC15	∼ 2 000 000	1.24	6.13
MolCLR	PubChem	∼ 10 000 000	0.71	4.68
G_Motif	ZINC15 and ChEMBL	∼ 11 000 000	NA**	NA**
MGSSL	ZINC15	∼ 250 000	**0.03**	**0.21**
HiMol	ZINC15	∼ 250 000	**0.03**	**0.21**
KPGT	ChEMBL29	∼ 2 000 000	12.89	81.67
KGG (*ours*)	ZINC15	∼ 250 000	**0.03**	**0.21**

**The pre-training datasets employed by these methods are* non-molecular*; they consist of paper-citation networks and Amazon product-review graphs.*

***The pre-training dataset referenced by the authors is unavailable; the download link supplied in the paper is no longer accessible.*

Table S12: Dataset sizes and processing times used in this work.

Phase	Dataset	Data points	Time	Unit
Pre-training	ZINC15	249 456	11.87	h
	ChEMBL29	2 084 715	109.23	h
Fine-tuning	BACE	1 513	4.90	min
	BBBP	2 053	4.88	min
	Tox21	7 831	13.02	min
	ToxCast	8 597	20.47	min
	SIDER	1 427	5.02	min
	ClinTox	1 484	4.21	min
	ESOL	1 128	2.99	min
	FreeSolv	642	1.76	min
	Lipophilicity	4 200	10.70	min
	QM7	6 834	7.72	min
	QM8	21 786	22.01	min
	QM9	133 885	138.36	min
Inference	BACE	1 513	996 ± 129	μs
	BBBP	2 053	831 ± 108	μs
	Tox21	7 831	65 ± 8	μs
	ToxCast	8 597	1 ± 0	μs
	SIDER	1 427	57 ± 6	μs
	ClinTox	1 484	388 ± 51	μs
	ESOL	1 128	673 ± 156	μs
	FreeSolv	642	1163 ± 924	μs
	Lipophilicity	4 200	798 ± 86	μs
	QM7	6 834	436 ± 115	μs
	QM8	21 786	25 ± 10	μs
	QM9	133 885	30 ± 10	μs

2 Datasets and benchmarks

2.1 Datasets

Knowledge-Guided Graph (`KGG`) is a cutting-edge self-supervised learning framework that employs a two-stage training process. Initially, the model is pre-trained on a large-scale dataset to develop robust and generalizable representations. Subsequently, it is fine-tuned on various downstream tasks to optimize its performance for specific applications. Detailed descriptions of all datasets utilized in this study are provided in the following sections.

Pre-training dataset For the pre-training phase, 250,000 unlabeled molecules were drawn from the publicly available ZINC15 database.[1] ZINC15 is widely employed for virtual screening workflows and is broadly adopted in drug discovery and pharmacophore-based analyses.

Fine-tuning datasets For the fine-tuning stage, we utilize datasets from MoleculeNet,[2] a comprehensive benchmark of over 700,000 compounds evaluated across diverse molecular properties. These properties are commonly grouped into four categories: *quantum mechanics*, *physical chemistry*, *biophysics*, and *physiology*. The individual datasets within MoleculeNet range from fundamental molecular properties to macroscopic physiological effects, providing varied and challenging prediction tasks. The detailed descriptions are provided below and summarized in Table S13.

1. Classification datasets

    - **BACE** contains quantitative binding data (IC_{50}) and binary labels for human beta-secretase 1 (BACE-1) inhibitors, encompassing 1,513 molecules.[3]

    - **BBBP (Blood-Brain Barrier Penetration)** comprises binary indicators of blood-brain barrier permeability for 2,053 compounds, which is critical for central nervous system drug development.[4]

    - **SIDER (Side Effect Resource)** documents adverse drug reactions (ADRs) across 27 MedDRA-classified organ systems for 1,427 FDA-approved drugs.[5]

- **ClinTox** comprises 1,484 drugs with annotations on clinical trial toxicity and FDA approval status, resulting in two binary classification tasks.[6]
- **Tox21** consists of 7,831 molecules labeled for 12 nuclear receptor signaling or stress response pathways, originating from the "Toxicology in the 21st Century" initiative.[7]
- **ToxCast** includes 8,597 compounds assessed in high-throughput toxicity assays, yielding 617 binary toxicity endpoints.[8]

2. Regression datasets

- **ESOL** provides 1,128 compounds with experimentally determined aqueous solubility values.[9]
- **Lipophilicity** contains 4,200 compounds with experimental log D (octanol-water distribution coefficients) values at pH = 7.4 extracted from `ChEMBL`,[10] representing an essential property for membrane permeability.
- **FreeSolv** covers 642 small molecules, detailing both experimental and computed hydration free energies,[11] partly used in the SAMPL blind prediction challenges.[12]
- **QM7** consists of 6,834 molecules selected from GDB-13, providing quantum-mechanically derived electronic properties and stable 3D configurations computed via density functional theory (DFT).[13]
- **QM8** contains 21,786 molecules (from GDB-17) with quantum mechanical properties (excited-state energies, electronic spectra) determined via TDDFT and CC2 methods.[14]
- **QM9** features 133,885 small organic molecules (up to nine heavy atoms), with extensive geometric, energetic, electronic, and thermodynamic properties calculated by DFT (B3LYP/6-31G(2df,p)).[15]

Table S13: Summary of fine-tuning datasets utilized in the study

Category	Dataset	Tasks	Task Type	Molecules	Metrics
Quantum Mechanics	QM7	1	Regression	6,834	MAE
	QM8	12	Regression	21,786	MAE
	QM9	12	Regression	133,885	MAE
Physical Chemistry	ESOL	1	Regression	1,128	RMSE
	FreeSolv	1	Regression	642	RMSE
	Lipophilicity	1	Regression	4,200	RMSE
Biophysics	BACE	1	Classification	1,513	ROC-AUC
Physiology	BBBP	1	Classification	2,053	ROC-AUC
	Tox21	12	Classification	7,831	ROC-AUC
	ToxCast	617	Classification	8,597	ROC-AUC
	SIDER	27	Classification	1,427	ROC-AUC
	ClinTox	2	Classification	1,484	ROC-AUC

2.2 Benchmarking models

In the context of evaluating performance on the MoleculeNet datasets,[2] we benchmarked our `KGG` model against thirteen state-of-the-art self-supervised learning (Self-Supervised Learning (SSL)) approaches. Detailed descriptions of each model are provided below:

– `GraphSAGE`[16] proposes a novel approach for aggregating information from neighboring nodes. In the pre-training stage, neighboring and non-neighboring node pairs are sampled as positive and negative examples, respectively, and SSL is performed based on edge prediction.

– `GPT-GNN`[17] implements probabilistic generative models to reconstruct graph structures and attributes. In this process, specific edges and node features are intentionally hidden, and the SSL model is trained to predict these obscured features and connections.

– `AttributeMask`[18] randomly hides specific edge attributes and utilizes a Graph Neural Networks (`GNNs`) to restore these concealed attributes.

- ContextPred[18] utilizes SSL framework built upon Graph Isomorphism Networks (GIN) by predicting the surrounding graph structures of specific motifs.

- InfoGraph[19] learns graph-level representations by maximizing mutual information between graph-level embeddings and substructures at various scales, including nodes and edges.

- MoCL[20] combines two distinct contrastive strategies: local contrast between nodes within a graph and global contrast between corresponding graph pairs.

- GraphLoG[21] pre-trains GIN through learning hierarchical prototypes based on graph embeddings.

- GraphCL[22] pre-trains GIN by maximizing mutual information between original graphs and their augmented counterparts. Graph augmentations are generated through node masking, edge addition, attribute masking, and subgraph insertion.

- JOAO[23] is an improved version of GraphCL, capable of adaptively selecting effective graph augmentation methods for contrastive learning.

- MolCLR[24] employs three graph augmentation methods: atom masking, bond deletion, and subgraph removal. SSL is conducted using contrastive learning.

- G-Motif[25] introduces a transformer-based message-passing network, framing motif prediction as a SSL signal.

- MGSSL[26] enhances motif construction rules, using GNNs as encoders for molecular graph representations. It predicts motifs according to a specified traversal order (either depth-first search or breadth-first search).

- HiMol[27] constructs hierarchical graphs that integrate multi-level SSL approaches.

Regarding the contamination analysis, we compare KGG with Knowledge-guided Pre-training of Graph Transformer (KPGT),[28] which transforms the original graphs into line graphs and

utilizes attention mechanisms for model training. We choose KPGT for comparison because it demonstrates higher performance relative to KGG. However, KPGT was pre-trained on two million molecules from the ChEMBL29 dataset,[29] whereas KGG was trained on only 250,000 sampled molecules from ZINC15.[1] This implementation demonstrates that KPGT artificially inflates its generalization performance by inadvertently including test instances in the pre-training process.

3 Methods Details

In this section, we present a comprehensive description of the KGG framework, with a particular emphasis on its motif decomposition and graph encoding methodologies.

Definition 3.1 (Graph). *An undirected graph $G = (V, E)$ is a set V vertices, which represent entities such as atoms, and a set E of edges, which represent connections like chemical bonds. Each edge is an unordered pair (v_i, v_j) of nodes in V.*

Definition 3.2 (Subgraph). *An subgraph $G' = (V', E')$ of a graph $G = (V, E)$ is a graph where $V' \subseteq V$ and $E' \subseteq E$. If $e = (v_i, v_j)$ is in E', then v_i, v_j must both be in V'.*

3.1 Motif Decomposition

Definition 3.3 (Motif Decomposition). *Let $G = (V, E)$ be a molecular graph, where V is the set of atoms and E is the set of chemical bonds. A* motif decomposition *of G is a partition*

$$\mathcal{M} \;=\; \{\, M_1, M_2, \dots, M_k \,\},$$

where each motif M_i is a subgraph $G_i = (V_i, E_i) \subseteq G$ that represents a chemically meaningful substructure, and the motifs are pairwise vertex– and edge—disjoint:

$$V_i \cap V_j = \varnothing, \quad E_i \cap E_j = \varnothing, \qquad \forall\, i \neq j.$$

Motif extraction proceeds in two sequential stages. First, all bonds that satisfy the BRICS cleavage rules[30] are removed, thereby partitioning the molecular graph into edge-disjoint BRICS fragments. Second, within each BRICS fragment, the ring-condensation decomposition procedure[27] is invoked to extract every minimal, non-overlapping ring, yielding the final set of chemically significant motifs. Algorithm S1 provides a concise pseudocode description of the entire workflow.

1. **BRICS Bond Cleavage**

Given a molecular graph $G = (V, E)$, the BRICS fragmentation procedure splits G along those bonds that satisfy the BRICS cleavage rules:[30]

(a) *Cleavable–bond predicate* : define $\beta : E \to \{\text{true}, \text{false}\}$ with $\beta(e) = \text{true}$ if bond $e \in E$ matches a `BRICS` pattern.

(b) *Collection of cleavable bonds* : $B = \{ e \in E \mid \beta(e) = \text{true} \}$.

(c) *Bond excision* : remove B to obtain $E' = E \setminus B$ and the residual graph $G' = (V, E')$.

(d) *Fragment enumeration* : let $\mathcal{C} = \big\{C_1 = (V_1, E_1), \ldots, C_k = (V_k, E_k)\big\}$ be the connected components of G'; each C_i is a `BRICS` fragment.

(e) *Output* : the set $\mathcal{C}$ constitutes the `BRICS`-based partition of G.

2. **Ring-Condensation Decomposition inside each BRICS Fragment**

For every `BRICS` fragment $C = (V_C, E_C) \in \mathcal{C}$:

(a) *Minimal-ring predicate* : Let $\mathcal{S}(C)$ be the collection of simple cycles in C. Define

$$\psi \; : \; \mathcal{S}(C) \; \longrightarrow \; \{\text{true}, \text{false}\},$$

where $\psi(R) = \text{true}$ if R is an *isolated ring*, i.e. R shares no edges with any other cycle in C. Such rings represent the individual members of any fused or condensed system.

(b) *Extraction of non-overlapping rings* : Collect every isolated ring,

$$\mathcal{R}(C) = \{ R \in \mathcal{S}(C) \mid \psi(R) = \text{true} \}.$$

By construction, the rings in $\mathcal{R}(C)$ are pairwise edge-disjoint.

(c) *Residual substructures*: Excise the edges of all rings in $\mathcal{R}(C)$,

$$E_C^{\text{res}} \;=\; E_C \setminus \bigcup_{R\in\mathcal{R}(C)} E(R),$$

and compute the connected components of the residual graph $C^{\text{res}} = (V_C, E_C^{\text{res}})$; denote this set by $\mathcal{Q}(C)$.

The complete motif contribution of C is the disjoint union

$$\mathcal{M}(C) \;=\; \mathcal{R}(C) \;\cup\; \mathcal{Q}(C),$$

where $\mathcal{R}(C)$ contains all individual, non-overlapping rings obtained by *cutting* the fused system, and $\mathcal{Q}(C)$ comprises the remaining fragments that persist once those rings have been removed.

Output. The final motif set is

$$\boxed{\mathcal{M} \;=\; \bigcup_{C\in\mathcal{C}} \mathcal{M}(C)}$$

and comprises (i) all minimal, edge-disjoint rings, (ii) the residual fragments obtained after ring removal, and/or (iii) any `BRICS` fragments that contained no condensed rings ($\mathcal{R}(C) = \varnothing$). By construction, the motifs in $\mathcal{M}$ are pairwise vertex- and edge-disjoint, and their union reconstructs the original vertex set V.

Algorithm S1 MotifDecomposition(G)

Require: Molecular graph $G = (V, E)$
Ensure: Motif set $\mathcal{M}$

$\mathcal{M} \leftarrow \varnothing$ ▷ final motif set
/* Stage 1 – BRICS Bond Cleavage */
$B \leftarrow \{\, e \in E \mid \beta(e) = \text{true} \,\}$ ▷ a set of cleavable bonds based on BRICS rules
$E' \leftarrow E \setminus B$ ▷ remove all cleavable bonds
$G' \leftarrow (V, E')$ ▷ the residual graph
$\mathcal{C} \leftarrow$ConnectedComponents(G') ▷ BRICS fragments
/* Stage 2 – Ring-Condensation Decomposition */
for all $C = (V_C, E_C) \in \mathcal{C}$ **do**
  $\mathcal{R} \leftarrow \varnothing$ ▷ isolated rings in C
  $\mathcal{S} \leftarrow$SimpleCycles(C)
  for all $R \in \mathcal{S}$ **do**
    if $\psi(R) = \text{true}$ **then** ▷ isolated ring test
      $\mathcal{R} \leftarrow \mathcal{R} \cup \{R\}$
    end if
  end for
  $E_C^{\text{res}} \leftarrow E_C \setminus \big(\cup_{R \in \mathcal{R}} E(R)\big)$
  $C^{\text{res}} \leftarrow (V_C, E_C^{\text{res}})$
  $\mathcal{Q} \leftarrow$ConnectedComponents(C^{res})
  $\mathcal{M} \leftarrow \mathcal{M} \cup \mathcal{R} \cup \mathcal{Q}$
end for
return $\mathcal{M}$

3.2 Molecular Encoding

In this work, a molecule is represented as a *hierarchical* graph

$$\mathcal{M} \;=\; \Big(\tilde{G} \;=\; (\tilde{V}, \tilde{E}),\ \{\mathbf{x}_\nu\}_{\nu \in \tilde{V}},\ \{\mathbf{x}_{\nu\mu}\}_{(\nu,\mu) \in \tilde{E}}\Big),$$

where

$$\tilde{V} \;=\; V \;\cup\; V_m \;\cup\; V_g, \quad \tilde{E} \;=\; E \;\cup\; E_m \;\cup\; E_g.$$

The sets V, V_m, and V_g denote atomic vertices, motif vertices, and supernode vertices, respectively, while E, E_m, and E_g represent the corresponding edges among these vertices. For each vertex $v \in \tilde{V}$, we define a node feature vector $\mathbf{x}_v \in \mathbb{R}^{d_{\text{node}}}$. Similarly, for each edge $(v_i, v_j) \in \tilde{E}$, we define an edge feature vector $\mathbf{x}_{v_i v_j} \in \mathbb{R}^{d_{\text{edge}}}$. The dimensions d_{node} and d_{edge}

are determined by the employed encoding scheme, as detailed in the following sections and summarized in Table S16.

3.2.1 Orbital-level Encoding Algorithm

All feature descriptors are computed entirely algorithmically from the molecular graph. We employ `RDKit` exclusively for SMILES parsing and to extract basic atom and bond properties; all higher-level orbital and bond descriptors are generated by our proprietary routines. Here, we outline the internal functions used to derive orbital-level features, which build upon RDKit's low-level APIs to calculate atom degree, implicit hydrogen counts, hybridization state, and corresponding VSEPR, inspired orbital occupancy vectors. A high-level description of this featurization is provided in the manuscript, while the detailed mathematical formulation is available in the Supporting Information. The full implementation can be found in our public GitHub repository: https://github.com/ThinhUMP/KGGraph/tree/main.

For each atom we first compute the number of surrounding atoms (including implicit hydrogens):

$$N_{\text{neigh\_count}} = \texttt{get\_degree(atom)} + \texttt{get\_total\_num\_hs(atom)},$$

and retrieve the hybridization label once, which is mapped to a maximum bonding capacity $B_{\max}$ via the lookup in Table S14. The number of lone pairs then follows as

$$N_{\text{lp}} = B_{\max} - N_{\text{neigh\_count}}.$$

Occupied s, p, and d orbital counts are obtained by indexing the dictionary in Table S15 with the key $(N_{\text{neigh\_count}}, N_{\text{lp}})$, yielding the five-entry vector

$$[\#s,\ \#p,\ \#d,\ N_{\text{neigh\_count}},\ N_{\text{lp}}].$$

If a key is absent, a zero vector $[0,0,0,0,0]$ is used.

Table S14: Maximum bonding capacity per hybridization state used to compute $N_{\mathrm{lp}} = B_{\max} - N_{\mathrm{neigh\_count}}$.

Hybridization	$B_{\max}$ (neigh_counts incl. H)
s	1
sp^2	2
sp^3	3
sp^3	4
sp^3d	5
sp^3d^2	6

Table S15: Lookup table for atom hybridization features. Each entry returns $[\#s, \#p, \#d, N_{\mathrm{neigh\_count}}, N_{\mathrm{lp}}]$ for the given $(N_{\mathrm{neigh\_count}}, N_{\mathrm{lp}})$ key.

$(N_{\mathrm{neigh\_count}}, N_{\mathrm{lp}})$	s	p	d	$N_{\mathrm{neigh\_count}}$	N_{lp}	Hybrid	Example
(1,0)	1	0	0	1	0	s	Na in NaI
(0,0)	1	0	0	0	0	s	Zn^{2+}
(0,1)	1	0	0	0	1	s	H^-
(1,1)	1	1	0	1	1	sp	N of HCN
(2,0)	1	1	0	2	0	sp	C≡C
(0,2)	1	1	0	0	2	sp	Cr^{3+}
(2,1)	1	2	0	2	1	sp^2	N of pyrimidine
(1,2)	1	2	0	1	2	sp^2	O of C=O
(3,0)	1	2	0	3	0	sp^2	C of C=C
(0,3)	1	2	0	0	3	sp^2	Fe^{2+}
(1,3)	1	3	0	1	3	sp^3	R-X (halogen)
(2,2)	1	3	0	2	2	sp^3	O of R-O-R′
(3,1)	1	3	0	3	1	sp^3	N of NR_3
(4,0)	1	3	0	4	0	sp^3	C of CR_4
(0,4)	1	3	0	0	4	sp^3	X^- (halogen)
(6,-2)	1	3	0	6	0	sp^3	SbH_6^{3+}
(2,3)	1	3	1	2	3	sp^3d	Co complex
(3,2)	1	3	1	3	2	sp^3d	—
(4,1)	1	3	1	4	1	sp^3d	—
(5,0)	1	3	1	5	0	sp^3d	PCl_5
(0,5)	1	3	1	0	5	sp^3d	Ag complex
(6,-1)	1	3	1	6	0	sp^3d	AlH_3 complex
(4,2)	1	3	2	4	2	sp^3d_2	—
(2,4)	1	3	2	2	4	sp^3d_2	$PdCl_2$
(3,3)	1	3	2	3	3	sp^3d_2	Dy complex
(5,1)	1	3	2	5	1	sp^3d_2	—
(1,5)	1	3	2	1	5	sp^3d_2	CuI
(6,0)	1	3	2	6	0	sp^3d_2	S of SF_6

Bond-level features are computed analogously by our internal routines. For each bond we obtain the effective bond order $o = \texttt{get\_bond\_order(bond)}$ (Single = 1, Double = 2, Triple = 3, Aromatic = 1.5) and define

$$\sigma = \begin{cases} 1, & o \geq 1, \\ 0, & o < 1, \end{cases} \qquad \pi = \max\bigl(0,\, o-1\bigr), \qquad c = \texttt{is\_conjugated(bond)} \in \{0,1\}.$$

Here, `is_conjugated(bond)` returns a binary indicator of aromatic or otherwise delocalized π bonding. The scalars σ, π, and c are concatenated to form the bond feature vector $[\sigma,\ \pi,\ c]$. Finally, per-atom orbital vectors and per-bond vectors are concatenated to yield the complete molecular representation used in Figure 1b of the manuscript.

3.2.2 Node Feature Vector

When $v \in V$ corresponds to an actual atom, the associated node feature vector is given by

$$\mathbf{x}_v = \bigl(Z_v,\ d(v),\ s_v,\ p_v,\ d_v,\ \mathrm{adj}(v),\ \mathrm{lp}(v)\bigr),$$

where:

$$\begin{aligned}
Z_v &\in \{0,1,2,\ldots,118,119,120\}, && \text{(atomic number or an encoded identifier)},\\
d(v) &\in \{0,1,\ldots,10\}, && \text{(degree: total count of bonds incident on } v\text{)},\\
s_v &\in \{0,1\}, && \text{(}s\text{-orbital count in the hybridization of } v\text{)},\\
p_v &\in \{0,1,2,3\}, && \text{(}p\text{-orbital count in the hybridization of } v\text{)},\\
d_v &\in \{0,1,2\}, && \text{(}d\text{-orbital count in the hybridization of } v\text{)},\\
\mathrm{adj}(v) &= \sum_{v_i,v_j\in V} \mathbb{I}\big((v_i,v_j)\in E\big) \in \{0,\ldots,6\}, && \text{(number of atoms adjacent to } v\text{)},\\
\mathrm{lp}(v) &\in \{0,\ldots,6\}, && \text{(number of lone pairs on } v\text{)}.
\end{aligned}$$

Here, $\mathbb{I}(\cdot)$ denotes the indicator function, returning 1 if its argument is true and 0 otherwise.

3.2.3 Edge Feature Vector

For each edge $(v_i, v_j) \in E$, the edge feature vector is expressed as

$$\mathbf{x}_{v_i v_j} = \big(\mathrm{type}(v_i,v_j),\ \mathrm{cycle}(v_i,v_j),\ \sigma_{v_i v_j},\ \pi_{v_i v_j},\ \delta_{v_i v_j}\big),$$

where:

$$\begin{aligned}
\mathrm{type}(v_i,v_j) &\in \mathcal{T}, && \text{(bond type: single, double, triple, aromatic, others)},\\
\mathrm{cycle}(v_i,v_j) &\in \{0,1\}, && \text{(indicator that the pair } \{v_i,v_j\} \text{ forms part of a ring)},\\
\sigma_{v_i v_j} &\in \{0,1\}, && \text{(presence or absence of a } \sigma\text{-bond component)},\\
\pi_{v_i v_j} &\in \{0,\ 0.5,\ 1,\ 2\}, && \text{(encoded strength of the } \pi\text{-bond component)},\\
\delta_{v_i v_j} &\in \{0,1\}, && \text{(indicator for extended conjugation)}.
\end{aligned}$$

The set $\mathcal{T}$ also comprises other connection types, including node–motif, motif–supernode, node–supernode, and node self-loops links.

Table S16: Summary of molecular encoding features. The symbol "various" in the table indicates multiple allowable types of bonds or connections (e.g., single, double, triple, aromatic, or other special connections to motifs or supernodes).

Type	Feature	Data Type	Allowed Values
**Node**	Z_v (Atomic number)	Integer	$\{0, 1, \ldots, 118, 119, 120\}^*$
	$d(v)$ (Degree)	Integer	$\{0, 1, \ldots, 10\}$
	s_v (s orbital)	Integer	$\{0, 1\}$
	p_v (p orbitals)	Integer	$\{0, 1, 2, 3\}$
	d_v (d orbitals)	Integer	$\{0, 1, 2\}$
	$\mathrm{adj}(v)$ (Adjacent atoms)	Integer	$\{0, \ldots, 6\}$
	$\mathrm{lp}(v)$ (Lone pairs)	Integer	$\{0, \ldots, 6\}$
**Edge**	$\mathrm{type}(v_i, v_j)$ (Bond type)	Integer	various
	$\mathrm{cycle}(v_i, v_j)$ (Cycle indicator)	Integer	$\{0, 1\}$
	$\sigma_{v_i v_j}$ (Sigma bond)	Integer	$\{0, 1\}$
	$\pi_{v_i v_j}$ (Pi bond strength)	Float	$\{0, 0.5, 1, 2\}$
	$\delta_{v_i v_j}$ (Conjugation indicator)	Integer	$\{0, 1\}$

* Here, 119 indicates a supernode and 120 indicates a motif.

3.3 KRG Encoders

Dimensional transformation in the KRG Encoder.

Node feature projection Let $H := \{h_v\}_{v \in \tilde{V}} \in \mathbb{R}^{|\tilde{V}|} \times 7$, where each row h_v stores the 7-dimensional attribute vector of node v and the index set $\tilde{V}$ spans *all* nodes in the hierarchical graph. This matrix is fed into a node-level MLP ϕ_n that projects the raw features to the hidden dimension $d = 512$:

$$\mathbf{H}_v^{(0)} = \phi_n(H) \in \mathbb{R}^{|\tilde{V}| \times d}.$$

Edge feature projection Similarly, define $\mathbf{X}_e := \{\mathbf{x}_e\}_{e \in \tilde{E}} \in \mathbb{R}^{|\tilde{E}|} \times 5$, where each row $\mathbf{x}_e$ stores the 5-dimensional attribute vector of edge e and the index set $\tilde{E}$ spans *all* edges

in the hierarchical graph. Passing $\mathbf{X}_e$ through an edge-level MLP ϕ_e yields the hidden representation

$$\mathbf{H}_e^{(0)} = \phi_e(\mathbf{X}_e) \ \in \ \mathbb{R}^{|\tilde{E}|\times d}, \qquad d = 512.$$

KRG encoder For each layer $\ell = 1, \ldots, L$ (with $L = 5$ in our implementation), the hidden node representation is updated via a `KRG` (GIN-style) block:

$$\mathbf{H}_v^{(\ell)} \ = \ \mathrm{GINConv}_\ell\big(\mathbf{H}_v^{(\ell-1)}, \mathbf{H}_e^{(0)}\big), \quad \mathbf{H}_v^{(\ell)} \in \mathbb{R}^{|\tilde{\mathcal{V}}|\times d}.$$

The hidden width $d = 512$ is kept *constant* throughout all five layers.

Graph read-out No explicit pooling is applied. The hierarchical graph is built with a dedicated *root* (super) node v_g that collects information from all other nodes, and motifs during message passing. Hence, its embedding after the last convolutional layer serves directly as the *graph-level* representation:

$$\mathbf{h}_G \ = \ \mathbf{H}_v^{(L)}[v_g, :] \ \in \ \mathbb{R}^{1\times d}, \qquad d = 512.$$

Prediction head The graph-level vector $\mathbf{h}_G$ is fed into a two-layer multilayer perceptron $\psi(\cdot)$, which projects the representation to the task space,

$$\hat{\mathbf{y}} = \psi(\mathbf{h}_G) \ \in \ \mathbb{R}^{1\times T},$$

where T denotes the number of downstream prediction targets.

3.4 Ablation Study

An ablation study is an analytical method widely utilized in the fields of machine learning and Artificial Intelligence (AI) to evaluate the significance of individual components or factors within a model. By systematically removing or modifying various elements of the model

and subsequently observing changes in its performance, one can effectively identify which components contribute most significantly to the overall model efficacy.[31] Based on this principle, this study proposes several variants of the KGG model to demonstrate the importance of orbital-oriented pre-training tasks and the two knowledge vectors: hybridization and bond type.

Self-Supervised Learning Variants of KGG

The objective of this implementation is to demonstrate that the KGG model benefits from the pre-training process. Therefore, this study introduces variants of KGG at three different levels of self-supervised learning: (i) no pre-training, (ii) partial pre-training, and (iii) full pre-training. These variants are detailed in the Table S17.

Table S17: KGG variants in the pre-training stage

Model	Full Name	Implementation Method
KGGwoPre	Knowledge-Guided Graph without Pre-training	The datasets from MoleculeNet are fine-tuned directly without undergoing any pre-training.
KGGHyBowoPre	Knowledge-Guided Graph with Hybridization and Bond type yet no Pre-training tasks	The KGG model features hybridization and bond type vectors. However, the pre-training Knowledge Self-Supervised Pre-training (KSSP) only performs three tasks: reconstructing the adjacency matrix, and predicting the number of atoms and bonds in a molecule.

Knowledge Vectors' importance

The goal of this section is to demonstrate the effectiveness of the hybridization and bond type vectors in molecular representation, in comparison to (a) a model that does not implement this feature creation approach, and (b) the traditional one-hot encodings. The study progressively removes the hybridization and bond type vectors from the KGG model. Concurrently, a one-hot model is constructed by replacing the knowledge vectors with corresponding one-hot vectors. Table S18 presents details of each variant and their

respective training procedures.

Table S18: KGG variants with different feature generation methods

**Model**	**Full Name**	**Implementation Method**
`KGGwoHybri`	Knowledge-Guided Graph without Hybridization	The `KGG` model removes the hybridization vector along with the five pre-training tasks related to this vector.
`KGGwoBondType`	Knowledge-Guided Graph without Bond type	The `KGG` model removes the bond type vector along with the three pre-training tasks related to this vector.
`KGGwoHyBo`	Knowledge-Guided Graph without Hybridization and Bond type	The `KGG` model removes both the hybridization and bond type vectors along with the eight pre-training tasks associated with these vectors.
`KGGonehot`	Knowledge-Guided Graph one-hot	The `KGG` model removes the hybridization and bond type vectors, replacing them with one-hot vectors. Concurrently, the pre-training tasks related to two knowledge vectors are replaced with tasks predicting hybridization and bond types using the Cross Entropy Loss (`CELoss`).

3.5 Motif Representation Visualization

We visualize motif-level representations by the following steps. The protocol described below was applied to `KGG` that had been pre-trained on $\sim 250\,\mathrm{k}$ molecules from the `ZINC15` corpus and fine-tuned on the classification benchmarks `BACE`, `BBBP`, and `ClinTox` as well as all six regression datasets (`ESOL`, `FreeSolv`, `Lipophilicity`, `QM7`, `QM8`, and `QM9`).

(1) **Motif-level representation extraction.** For each molecule M in the downstream fine-tuning set, pass it through the `KRG` encoder to obtain the set of motif embeddings

$$\mathbf{H}_m = \{\mathbf{m}_s \mid s \in V_m\} \subseteq \mathbb{R}^{|V_m| \times d}, \qquad d = 512,$$

where V_m is the index set of motifs in M.

(2) **Global molecular representation.** Aggregate the motif embeddings by summing over the first dimension:

$$\mathbf{h}_g = \sum_{s \in V_m} \mathbf{m}_s \in \mathbb{R}^d.$$

(3) **Dimensionality reduction.** Collect the global vectors $\{\mathbf{h}_g\}_{M=1}^{N}$ and project them onto $\mathbb{R}^2$ using a manifold-learning algorithm (`t-SNE`), retaining the ground-truth class labels of each molecule M for later use.

(4) **Visualisation and qualitative assessment.** Render a two-dimensional scatter plot, coloring (or otherwise marking) each point by its ground-truth label. Examine the resulting plot for label-consistent clusters reveals about the discriminative capacity of the learned motif representations.

3.6 Experimental configuration

3.6.1 Pre-training Parameters

The `KSSP` and `KRG` models utilized the `Adam` optimizer and were pre-trained with the hyper-parameters presented in Table S19 until convergence.

Table S19: Hyperparameters used for pre-training the `KSSP` and `KRG` models.

**Hyperparameter**	**Value**	**Description**
Epochs	60	Number of passes through the dataset
Learning rate	0.001	Model learning rate
Weight decay	0	Penalty coefficient to prevent overfitting
`KRG` Dropout	0.5	Randomly ignores a proportion of neurons during training
`KSSP` Dropout	0.2	Randomly ignores a proportion of neurons during training
Batch size	32	Number of samples used per gradient update
Number of workers	10	Number of CPUs for loading data in training
Embedding size	512	Dimensionality of output vector in `KRG` model

3.6.2 Fine-tuning Process

The hyperparameters used for fine-tuning, corresponding to each dataset, are shown in Table S20. Detailed descriptions of these hyperparameters are provided in Table S21.

Table S20: Hyperparameters for the Fine-tuning Process

Dataset	lr_feat	lr_pred	decay	dropout	batch_size	epoch	dim
BACE	1e-3	1e-3	1e-7	0.6	32	100	512
BBBP	5e-4	1e-3	0	0.8	32	100	512
Tox21	5e-4	1e-3	0	0.7	32	100	512
ToxCast	1e-3	1e-3	1e-7	0.7	32	100	512
SIDER	5e-4	1e-3	2e-4	0.6	32	100	512
ClinTox	1e-3	1e-3	0	0.6	32	100	512
ESOL	1e-3	1e-3	0	0.7	32	100	512
FreeSolv	1e-3	1e-3	0	0.5	32	100	512
Lipophilicity	5e-4	5e-4	0	0.5	32	100	512
QM7	1e-3	1e-3	1e-7	0.6	32	100	512
QM8	1e-3	1e-3	0	0.5	32	100	512
QM9	1e-3	1e-3	1e-7	0.5	32	100	512

Table S21: Definitions of fine-tuning hyperparameters

Hyperparameter	Description
lr_feat	Learning rate for the `KRG` decoder
lr_pred	Learning rate for the predictive `MLP` layer
decay	Regularization coefficient to prevent overfitting
dropout	Proportion of neurons randomly deactivated during training
batch_size	Number of samples used for each parameter update step
epoch	Number of iterations through the dataset
dim	Dimensionality of the output vector in the `KRG` model

3.7 Masking Experiment

Let the hierarchical graph be $\tilde{G} = (\tilde{V}, \tilde{E})$ with $\tilde{V} = V \cup V_m \cup V_g$ and $\tilde{E} = E \cup E_m \cup E_g$ (see Section 4.1 for details). Here, V and E denote the *atom-level* nodes and their chemical bonds. During masking we perturb *only* this atom layer; all *motif* nodes V_m, motif–atom edges E_m, the supernode V_g, and its edges E_g are left untouched, so that the hierarchical

graph structure of $\tilde{G}$ stays connected even under heavy masking. Two defined rates control the amount of perturbation:

$$p_n \in [0,1] \quad \text{(atom-masking rate)}, \qquad p_e \in [0,1] \quad \text{(bond-masking rate)}.$$

1. **Atom-feature masking.** Pick at random $\lceil p_n\,|V|\rceil$ atoms from V and replace each selected feature vector $h_v \in \mathbb{R}^d$, where $d = 7$, by the masked vector $\boldsymbol{\delta}_{\text{mask}} = (121, 0, 0, 0, 0, 0, 0)$. 121 is an identified element for masked nodes.

2. **Edge masking.** Pick at random $\lceil p_e\,|E|\rceil$ edges from E to obtain E_{mask}; removing these edges yields the sparsified edge set $E' = E \setminus E_{\text{mask}}$, the updated adjacency matrix A', and the revised edge-feature matrix $\mathbf{X}'_e \in \mathbb{R}^{|E'|\times 5}$ (each bond has a 5-dimensional feature vector).

Table S22: Performance of `KGG` under random masking of orbital features of ***nodes (remove orbital features in hybridization vectors)***. Classification tasks are reported with ROC-AUC (↑); regression tasks with RMSE or MAE (↓). The symbol (↑) denotes that higher values indicate better performance. In contrast, the symbol (↓) denotes that lower values indicate better performance.

**Task Type**	**Dataset**	**Metric**	**0 %**	**10 %**	**20 %**	**30 %**	**40 %**	**50 %**
Classification	BACE	ROC-AUC ↑	86.3	79.8	77.0	77.4	80.8	77.5
Classification	BBBP	ROC-AUC ↑	72.5	70.9	69.7	68.7	67.1	65.1
Classification	Tox21	ROC-AUC ↑	75.6	73.1	73.3	73.6	71.8	72.6
Classification	ToxCast	ROC-AUC ↑	66.4	65.4	65.3	62.6	63.1	63.3
Classification	SIDER	ROC-AUC ↑	64.9	61.3	60.9	59.1	58.9	59.2
Classification	ClinTox	ROC-AUC ↑	87.3	72.2	80.4	71.3	64.9	71.5
Classification	Average	ROC-AUC ↑	75.5	70.5	71.1	68.8	67.8	68.2
Regression	ESOL	RMSE ↓	0.944	1.122	1.062	1.148	1.338	1.512
Regression	FreeSolv	RMSE ↓	0.731	0.797	0.792	0.761	0.974	0.948
Regression	Lipo	RMSE ↓	0.665	0.856	0.930	0.862	1.012	0.955
Regression	Average	RMSE ↓	0.780	0.925	0.928	0.924	1.108	1.138
Regression	QM7	MAE ↓	77.6838	96.127	98.432	99.925	103.958	118.244
Regression	QM8	MAE ↓	0.019	0.021	0.022	0.023	0.024	0.025
Regression	QM9	MAE ↓	3.526	5.015	5.409	6.663	7.865	9.260
Regression	Average	MAE ↓	27.076	33.721	34.621	35.537	37.282	42.510

Table S23: Performance of KGG under random masking of orbital features of ***edges (remove bond type vectors)***. Classification tasks are reported with ROC-AUC (↑); regression tasks with RMSE or MAE (↓). The symbol (↑) denotes that higher values indicate better performance. In contrast, the symbol (↓) denotes that lower values indicate better performance.

**Task Type**	**Dataset**	**Metric**	**0 %**	**10 %**	**20 %**	**30 %**	**40 %**	**50 %**
Classification	BACE	ROC-AUC ↑	86.3	80.4	80.8	75.3	77.2	73.2
Classification	BBBP	ROC-AUC ↑	72.5	66.5	68.2	67.9	66.4	66.4
Classification	Tox21	ROC-AUC ↑	75.6	74.7	74.2	74.5	73.4	72.7
Classification	ToxCast	ROC-AUC ↑	66.4	66.7	64.8	65.5	63.8	64.0
Classification	SIDER	ROC-AUC ↑	64.9	61.9	60.8	57.4	59.5	59.1
Classification	ClinTox	ROC-AUC ↑	87.3	78.4	66.9	77.3	76.3	73.4
Classification	Average	ROC-AUC ↑	75.5	71.4	69.3	69.7	69.4	68.1
Regression	ESOL	RMSE ↓	0.944	0.990	1.034	0.960	1.046	1.063
Regression	FreeSolv	RMSE ↓	0.731	0.773	0.736	0.733	0.692	0.698
Regression	Lipo	RMSE ↓	0.665	0.772	0.843	0.893	0.875	0.915
Regression	Average	RMSE ↓	0.780	0.845	0.871	0.862	0.871	0.892
Regression	QM7	MAE ↓	77.6838	86.559	82.464	84.228	77.667	82.348
Regression	QM8	MAE ↓	0.019	0.020	0.020	0.020	0.021	0.021
Regression	QM9	MAE ↓	3.526	4.917	6.031	5.366	5.108	5.328
Regression	Average	MAE ↓	27.076	30.499	29.505	29.871	27.599	29.232

Table S24: Performance of KGG under random masking of orbital features of both ***nodes and edges***. Classification tasks are reported with ROC-AUC (↑); regression tasks with RMSE or MAE (↓). The symbol (↑) denotes that higher values indicate better performance. In contrast, the symbol (↓) denotes that lower values indicate better performance.

**Task Type**	**Dataset**	**Metric**	**0 %**	**10 %**	**20 %**	**30 %**	**40 %**	**50 %**
Classification	BACE	ROC-AUC ↑	86.3	75.3	75.4	76.8	75.0	73.7
Classification	BBBP	ROC-AUC ↑	72.5	64.3	64.9	67.2	65.6	66.5
Classification	Tox21	ROC-AUC ↑	75.6	72.5	72.5	71.3	70.9	69.6
Classification	ToxCast	ROC-AUC ↑	66.4	65.1	64.1	62.2	60.9	60.5
Classification	SIDER	ROC-AUC ↑	64.9	62.0	58.6	59.0	56.2	56.0
Classification	ClinTox	ROC-AUC ↑	87.3	65.6	60.7	72.2	64.1	77.9
Classification	Average	ROC-AUC ↑	75.5	67.5	66.0	68.1	65.5	67.4
Regression	ESOL	RMSE ↓	0.944	1.129	1.104	1.197	1.655	1.615
Regression	FreeSolv	RMSE ↓	0.731	0.796	0.852	0.902	0.906	1.011
Regression	Lipo	RMSE ↓	0.665	0.872	0.994	1.016	1.047	1.027
Regression	Average	RMSE ↓	0.780	0.932	0.983	1.038	1.203	1.218
Regression	QM7	MAE ↓	77.6838	91.303	100.015	107.593	105.366	133.904
Regression	QM8	MAE ↓	0.019	0.021	0.022	0.026	0.027	0.028
Regression	QM9	MAE ↓	3.526	6.546	6.980	9.866	12.119	14.006
Regression	Average	MAE ↓	27.076	32.623	35.672	39.162	39.171	49.313

As detailed in Tables S22-S24, mean ROC-AUC over six classification tasks falls from 75.5 % to 68.2 % under 50 % node masking and to 68.1 % under 50 % edge masking, while the average RMSE across ESOL/FreeSolv/Lipo rises from 0.780 to 1.138 and 0.892, respectively; even simultaneous node + edge masking retains 67.4 % ROC-AUC and around 1.0 RMSE. These modest degradations indicate that KGG can discount substantial feature loss because its hierarchical message passing preserves motif and graph-level context and distributes chemical information across multiple channels. Future work will extend this analysis to additive noise and systematic biases and will employ Bayesian ensembling to quantify uncertainty under corrupted inputs.

References

(1) Sterling, T.; Irwin, J. J. ZINC 15–ligand discovery for everyone. *Journal of chemical information and modeling* **2015**, *55*, 2324–2337.

(2) Wu, Z.; Ramsundar, B.; Feinberg, E. N.; Gomes, J.; Geniesse, C.; Pappu, A. S.; Leswing, K.; Pande, V. MoleculeNet: a benchmark for molecular machine learning. *Chemical science* **2018**, *9*, 513–530.

(3) Subramanian, G.; Ramsundar, B.; Pande, V.; Denny, R. A. Computational modeling of β-secretase 1 (BACE-1) inhibitors using ligand based approaches. *Journal of chemical information and modeling* **2016**, *56*, 1936–1949.

(4) Martins, I. F.; Teixeira, A. L.; Pinheiro, L.; Falcao, A. O. A Bayesian approach to in silico blood-brain barrier penetration modeling. *Journal of chemical information and modeling* **2012**, *52*, 1686–1697.

(5) Kuhn, M.; Letunic, I.; Jensen, L. J.; Bork, P. The SIDER database of drugs and side effects. *Nucleic acids research* **2016**, *44*, D1075–D1079.

(6) Gayvert, K. M.; Madhukar, N. S.; Elemento, O. A data-driven approach to predicting successes and failures of clinical trials. *Cell chemical biology* **2016**, *23*, 1294–1301.

(7) Tox21 Data Challenge 2014. https://tripod.nih.gov/tox21/challenge/, 2014.

(8) Richard, A. M.; Judson, R. S.; Houck, K. A.; Grulke, C. M.; Volarath, P.; Thillainadarajah, I.; Yang, C.; Rathman, J.; Martin, M. T.; Wambaugh, J. F.; others ToxCast chemical landscape: paving the road to 21st century toxicology. *Chemical research in toxicology* **2016**, *29*, 1225–1251.

(9) Delaney, J. S. ESOL: estimating aqueous solubility directly from molecular structure. *Journal of chemical information and computer sciences* **2004**, *44*, 1000–1005.

(10) Gaulton, A.; Bellis, L. J.; Bento, A. P.; Chambers, J.; Davies, M.; Hersey, A.; Light, Y.; McGlinchey, S.; Michalovich, D.; Al-Lazikani, B.; others ChEMBL: a large-scale bioactivity database for drug discovery. *Nucleic acids research* **2012**, *40*, D1100–D1107.

(11) Mobley, D. L.; Guthrie, J. P. FreeSolv: a database of experimental and calculated hydration free energies, with input files. *Journal of computer-aided molecular design* **2014**, *28*, 711–720.

(12) Mobley, D. L.; Wymer, K. L.; Lim, N. M.; Guthrie, J. P. Blind prediction of solvation free energies from the SAMPL4 challenge. *Journal of computer-aided molecular design* **2014**, *28*, 135–150.

(13) Blum, L. C.; Reymond, J.-L. 970 million druglike small molecules for virtual screening in the chemical universe database GDB-13. *Journal of the American Chemical Society* **2009**, *131*, 8732–8733.

(14) Ramakrishnan, R.; Hartmann, M.; Tapavicza, E.; von Lilienfeld, O. A. Electronic spectra from TDDFT and machine learning in chemical space. *The Journal of Chemical Physics* **2015**, *143*, 084111.

(15) Ruddigkeit, L.; Van Deursen, R.; Blum, L. C.; Reymond, J.-L. Enumeration of 166 billion organic small molecules in the chemical universe database GDB-17. *Journal of chemical information and modeling* **2012**, *52*, 2864–2875.

(16) Hamilton, W.; Ying, Z.; Leskovec, J. Inductive Representation Learning on Large Graphs. Advances in Neural Information Processing Systems. 2017.

(17) Hu, Z.; Dong, Y.; Wang, K.; Chang, K.-W.; Sun, Y. GPT-GNN: Generative pre-training of graph neural networks. Proceedings of the 26th ACM SIGKDD international conference on knowledge discovery & data mining. 2020; pp 1857–1867.

(18) Hu*, W.; Liu*, B.; Gomes, J.; Zitnik, M.; Liang, P.; Pande, V.; Leskovec, J. Strategies for Pre-training Graph Neural Networks. International Conference on Learning Representations. 2020.

(19) Sun, F.-Y.; Hoffmann, J.; Verma, V.; Tang, J. Infograph: Unsupervised and semi-supervised graph-level representation learning via mutual information maximization. *arXiv preprint arXiv:1908.01000* **2019**,

(20) Sun, M.; Xing, J.; Wang, H.; Chen, B.; Zhou, J. MoCL: data-driven molecular fingerprint via knowledge-aware contrastive learning from molecular graph. Proceedings of the 27th ACM SIGKDD conference on knowledge discovery & data mining. 2021; pp 3585–3594.

(21) Xu, M.; Wang, H.; Ni, B.; Guo, H.; Tang, J. Self-supervised graph-level representation learning with local and global structure. International Conference on Machine Learning. 2021; pp 11548–11558.

(22) You, Y.; Chen, T.; Sui, Y.; Chen, T.; Wang, Z.; Shen, Y. Graph contrastive learning with augmentations. *Advances in neural information processing systems* **2020**, *33*, 5812–5823.

(23) You, Y.; Chen, T.; Shen, Y.; Wang, Z. Graph contrastive learning automated. International Conference on Machine Learning. 2021; pp 12121–12132.

(24) Wang, Y.; Wang, J.; Cao, Z.; Barati Farimani, A. Molecular contrastive learning of representations via graph neural networks. *Nature Machine Intelligence* **2022**, *4*, 279–287.

(25) Rong, Y.; Bian, Y.; Xu, T.; Xie, W.; Wei, Y.; Huang, W.; Huang, J. Self-supervised graph transformer on large-scale molecular data. *Advances in neural information processing systems* **2020**, *33*, 12559–12571.

(26) Zhang, Z.; Liu, Q.; Wang, H.; Lu, C.; Lee, C.-K. Motif-based graph self-supervised learning for molecular property prediction. *Advances in Neural Information Processing Systems* **2021**, *34*, 15870–15882.

(27) Zang, X.; Zhao, X.; Tang, B. Hierarchical molecular graph self-supervised learning for property prediction. *Communications Chemistry* **2023**, *6*, 34.

(28) Li, H.; Zhang, R.; Min, Y.; Ma, D.; Zhao, D.; Zeng, J. A knowledge-guided pre-training framework for improving molecular representation learning. *Nature Communications* **2023**, *14*, 7568.

(29) Gaulton, A.; Hersey, A.; Nowotka, M.; Bento, A. P.; Chambers, J.; Mendez, D.; Mutowo, P.; Atkinson, F.; Bellis, L. J.; Cibrián-Uhalte, E.; others The ChEMBL database in 2017. *Nucleic acids research* **2017**, *45*, D945–D954.

(30) Degen, J.; Wegscheid-Gerlach, C.; Zaliani, A.; Rarey, M. On the art of compiling and using'drug-like'chemical fragment spaces. *ChemMedChem* **2008**, *3*, 1503.

(31) Vishnusai, Y.; Kulakarni, T. R.; Sowmya Nag, K. Ablation of Artificial Neural Networks. Innovative Data Communication Technologies and Application. Cham, 2020; pp 453–460.
